# Organic Crystal‐MXene Composites as Temperature‐Tolerant Strain Sensors

**DOI:** 10.1002/advs.202522007

**Published:** 2026-03-30

**Authors:** Xuesong Yang, Lijie Wang, Shun Liu, Yuhan Zhang, Yongqiang Xu, Haixia Mei, Liang Li, Ying Tao, Quan‐Hong Yang, Panče Naumov, Hongyu Zhang

**Affiliations:** ^1^ State Key Laboratory of Supramolecular Structure and Materials College of Chemistry Jilin University Changchun P. R. China; ^2^ State Key Laboratory of Integrated Optoelectronics College of Electronic Science and Engineering Jilin University Changchun P. R. China; ^3^ Smart Materials Lab New York University Abu Dhabi Abu Dhabi UAE; ^4^ SAFIR Novel Materials Development Lab Sorbonne University Abu Dhabi Abu Dhabi UAE; ^5^ Nanoyang Group Tianjin Key Laboratory of Advanced Carbon and Electrochemical Energy Storage School of Chemical Engineering and Technology Tianjin University Tianjin P. R. China; ^6^ Center for Smart Engineering Materials NYUAD Research Institute New York University Abu Dhabi Abu Dhabi UAE; ^7^ Research Center for Environment and Materials Macedonian Academy of Sciences and Arts Skopje Macedonia; ^8^ Molecular Design Institute Department of Chemistry New York University New York USA

**Keywords:** electrical conductivity, flexible sensors, low temperature, MXenes, organic crystals

## Abstract

Flexible organic crystals offer significant opportunities for organic electronics; however, their potential applications in fields like flexible sensors are at present limited by poor charge transport that result in failure under reversible conditions. In this report, we propose a universal interfacing design strategy for the fabrication of versatile conductive MXene coatings on the surface of flexible organic crystals that preserve the electric conductivity of the MXenes while they also remain mechanically flexible and robust across a wide temperature range of nearly 300°C both above and below ambient temperature. This new sub‐class of hybrid materials based on adaptive organic crystals undergoes reversible changes in the crystal structure upon deformation, while they also engender stable electrical conductivity and retain deformation sensitivity from about −196°C to 100°C. A wireless sensing system based on these materials was constructed to monitor bionic finger movements that demonstrate the potential for human‐machine interfacing and medical monitoring applications. The structural stability of this and similar devices that utilize crystal‐MXene hybrid materials under non‐ambient temperatures provide an innovative solution to the current challenges with occasional failure of low‐temperature mechanical sensing systems in extreme environments, and specifically such that are used in lunar and deep‐space explorations.

## Graphical abstract

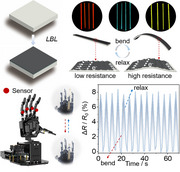

## 1 Introduction

Flexible electronic devices, being in intelligent sensing, smart interaction, and environmentally adaptive systems, are now driving the rapid developments of medical health monitoring [1, 2, 3], intelligent manufacturing [4, 5, 6], soft robotics [7, 8, 9], and sensing in extreme environments [10, 11, 12]. The efforts to develop flexible sensing materials with high sensitivity that are capable of stable operation over a wide temperature range under strenuous working conditions revolve around materials that are both structurally compliant and responsive to external stimuli. Flexible, dynamic or adaptive organic crystals―soft crystalline materials that are conceptually reminiscent of solid supramolecular machines―are rising in prominence for their ability to respond to some of these requirements by being able to perform complex functions, and these emerging capabilities are enhanced by behaviors similar to those of living organisms, allowing them to change shape and adapt, and these emerging capabilities are complemented with lifelike behaviors such as self‐healing [13, 14, 15, 16, 17, 18]. Unlike the “conventional” soft materials, whose flexibility relies on molecular chain entanglement or network breakage [19, 20], the flexibility of soft crystals originates mostly from the softness of their weak intermolecular interactions, with relatively modest energetic contribution coming from molecular flexibility; these characteristics endow the organic crystalline materials with the ability to maintain intact lattices under external influence, and even to encode structural memory [21, 22, 23, 24, 25, 26, 27, 28]. One of the consequences is an unprecedented low‐temperature elasticity, with some organic crystals remaining reversibly elastic and functional even at liquid nitrogen temperature (−196°C) [29, 30, 31, 32], a property that is of particular interest to devices such as low‐temperature flexible organic optical waveguides [33, 34, 35], rotational deflectors [36, 37] and amplified spontaneous emitters [38, 39, 40]. Despite the experimental evidence for the advantages of organic crystals for applications in non‐ambient conditions becoming increasingly abundant, the implementation in flexible devices is impeded by their limited charge transport [41, 42]. A second, equally relevant obstacle is related to the morphology of the crystals; there is very little maneuvering space when it comes to opportunities for molding or casting of these materials to fit the shape of the substrate, which leads to compatibility issues. This eventually results in loose interfaces with flexible substrates, a problem that may become critical during prolonged and reversible operation [43, 44].

Here, we propose a universal approach that addresses these challenges by being independent of the molecular and crystal structures of organic crystals, and is amenable to applications across diverse crystal morphologies and dimensions of naturally grown organic crystals. MXenes, particularly Ti_3_C_2_T_*x*_, are a class of 2D materials known for their high electrical conductivity, mechanical flexibility, and tunable surface chemistry, making them ideal for enhancing the conductivity of organic crystals [45, 46, 47, 48, 49, 50]. By employing the electrostatic layer‐by‐layer self‐assembly technique to construct conductive MXene networks on organic crystal surfaces, this method circumvents three critical issues: it provides a well‐defined, cohesive, and sustainable intermaterials interface, it accommodates various crystal sizes and habits, and it preserves the overall structural integrity upon prolonged and cyclic operation. These considerations led to the development of a new sub‐class of hybrid materials that deliver high sensitivity in electrical response (∼0.18 mm^−1^), stable performance across a wide temperature range for an organic material (−196°C to 100°C), reliable response to external stress, excellent mechanical flexibility, and long‐term structural stability, while they also remain elastic in cryogenic conditions. The experimental results show that the composite materials retain excellent resistance‐strain linearity even after multiple bending cycles, while maintaining their favorable electromechanical response accuracy at cryogenic temperatures. A wireless sensor prototype utilizing this material was fabricated as a proof‐of‐concept that is capable of precise bionic finger motion tracking, demonstrating their potential for human‐machine interfacing and clinical monitoring. This effective integration of MXenes in organic crystal‐based devices is likely to inspire further extreme‐temperature‐range sensing applications, while simultaneously establishing a new paradigm for thermally resilient flexible electronics. Compared with existing sensors and our prior work, this study introduces an organic crystal–MXene composite–based flexible sensor that combines high electrical conductivity over a wide temperature range (−196°C to 100°C) with excellent mechanical flexibility and strain sensitivity. This composite design extends the operational limits of previously reported sensors and provides a new strategy for sensing applications under extreme environmental conditions.

### 1.1 Design and Preparation of Flexible Organic Crystal‐MXene Composites

**FIGURE 1:**
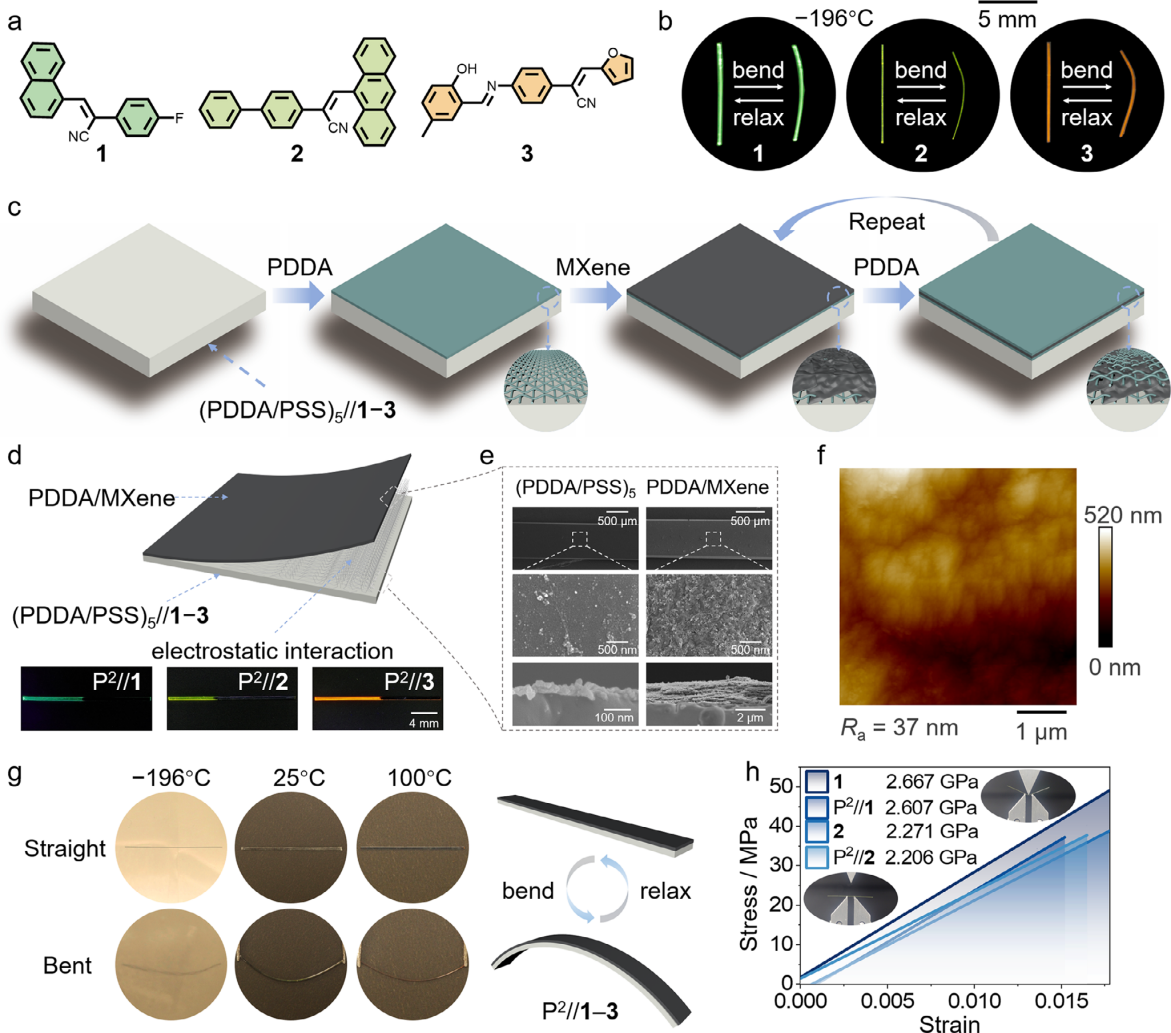
Preparation and characterization of flexible organic crystal‐MXene composite materials. (a) Molecular structures of **1**–**3**. (b) Photographs of reversible bending deformation of the organic crystals **1**–**3** at −196°C recorded under UV light. (c) Schematic diagram of the method used for preparation based on the electrostatic layer‐by‐layer self‐assembly. (d) Structural schematic diagram and photographs of organic crystal‐MXene composite materials. (e) Scanning electron microscopic images of the surface of (PDDA/PSS)_5_//**1** and P^2^//**1**. (f) Atomic force microscopic images of the surface of P^2^//**1**. (g) Photographs showing the bending of P^2^//**3** at three selected temperatures: −196°C, 25°C, and 100°C. (h) Stress‐strain curves of the native crystals **1**–**2** and the hybrids P^2^//**1**–**2**.

The combination of cryogenic elasticity of organic crystals and conductivity of MXenes described here is central to the development of flexible sensors that operate across wide temperature ranges. Since it does not depend on the actual chemical identity or the crystal structure, the protocol can be applied, in principle, to any flexible organic crystal. To introduce electrical conductivity, Ti_3_C_2_T_*x*_ MXene nanosheets were selected as the conductive component because of their high intrinsic electrical conductivity, excellent mechanical flexibility, and surface terminations that enable stable and uniform interfacial integration with polymer‐modified organic crystals. As a demonstration of its synthetic versatility, we selected three organic compounds with distinct molecular structures—(*E*)‐2‐(4‐fluorophenyl)‐3‐(naphthalen‐1‐yl)acrylonitrile, (*Z*)‐2‐([1,1′‐biphenyl]‐4‐yl)‐3‐(anthracen‐9‐yl)acrylonitrile, and (*Z*)‐3‐(furan‐2‐yl)‐2‐(4‐(((*E*)‐2‐hydroxy‐5‐methylbenzylidene)amino)phenyl)acrylonitrile (denoted as **1**–**3** in Figure 1a) (Figures S1–S3)—and grew them into slender, centimeter‐scale crystal planks (Figure S4a and Table S1) [51, 52, 53]. The crystals were selected for being elastic; upon application of stress to both of their ends, they undergo reversible deformation at both 25°C and −196°C without fracturing (Figure 1b and Figure S4b). These crystals were chosen as substrates because their centimeter‐scale dimensions, pronounced elastic bending behavior, and structural stability over a wide temperature range provide an ideal mechanical platform for constructing flexible conductive hybrids. To impart electrical conductivity, we developed a method for hybridization based on electrostatic interactions (Figure 1c). First, the pristine organic crystals **1**–**3** were immersed in an aqueous solution of poly(diallyldimethylammonium chloride) (PDDA, 1.0 mg mL^−1^) for 20 min to allow sufficient adsorption of the positively charged layer. The crystals were then gently rinsed with distilled water for 1 min to remove excess physically adsorbed species. Subsequently, the crystals were immersed in an aqueous solution of poly(sodium 4‐styrenesulfonate) (PSS, 1.0 mg mL^−1^) for another 20 min, followed by the same rinsing procedure. By repeating the PDDA/PSS deposition cycle five times, polymer‐coated crystals denoted as (PDDA/PSS)_5_//**1**–**3** were obtained. To introduce the conductive layer, the polymer‐coated crystals were first immersed again in the PDDA solution for 20 min to modify the surface charge, followed by rinsing. Subsequently, a MXene aqueous dispersion (10 mg mL^−1^) was uniformly deposited onto one of the two wide surfaces of the crystal using a syringe. After the coating was completely dried, the crystal was re‐immersed in the PDDA solution. This cycle of “MXene deposition–drying–PDDA modification” was repeated to yield the conductive and mechanically flexible hybrid composites PDDA/MXene//PDDA/PSS//**1**–**3** (for clarity, hereafter abbreviated as P^2^//**1**–**3**; Figure 1d and Tables S2−S4). The surfaces of (PDDA/PSS)_5_//**1** and P^2^//**1** were examined by scanning electron microscopy (SEM), which revealed that the MXene is deposited as densely packed and laterally continuous nanosheets on the crystal surface (Figure 1e). Complementary atomic force microscopy (AFM) measurements further confirmed that the MXene layer uniformly covers the crystal surface at the nanoscale (Figure 1f), demonstrating the formation of a continuous conductive network on the crystal surface. To investigate the role of PDDA/PSS surface modification, control experiments were conducted by depositing MXene nanosheets directly onto pristine organic crystals. As shown in Figure S6, without polymer modification the aqueous MXene dispersion showed poor wetting and nonuniform surface coverage, whereas the (PDDA/PSS)_5_‐modified crystals enabled continuous MXene deposition and conductive network formation. As seen by the simple bending experiments in Figure 1g, similar to the uncoated crystals, the hybrids P^2^//**3** were found to be elastic at three representative temperatures: −196°C, 25°C, and 100°C. The elastic moduli of P^2^//**1**–**2**, estimated from the stress‐strain curves were similar to those of the crystals **1**–**2** (Figure 1h). These results demonstrate that the surface functionalization does not have a significant effect on the mechanical properties, and the elastic properties of the organic crystals remain practically unaffected by both the coating and the temperature.

### 1.2 Conductivity and Structure Optimization of the Composite Materials

**FIGURE 2:**
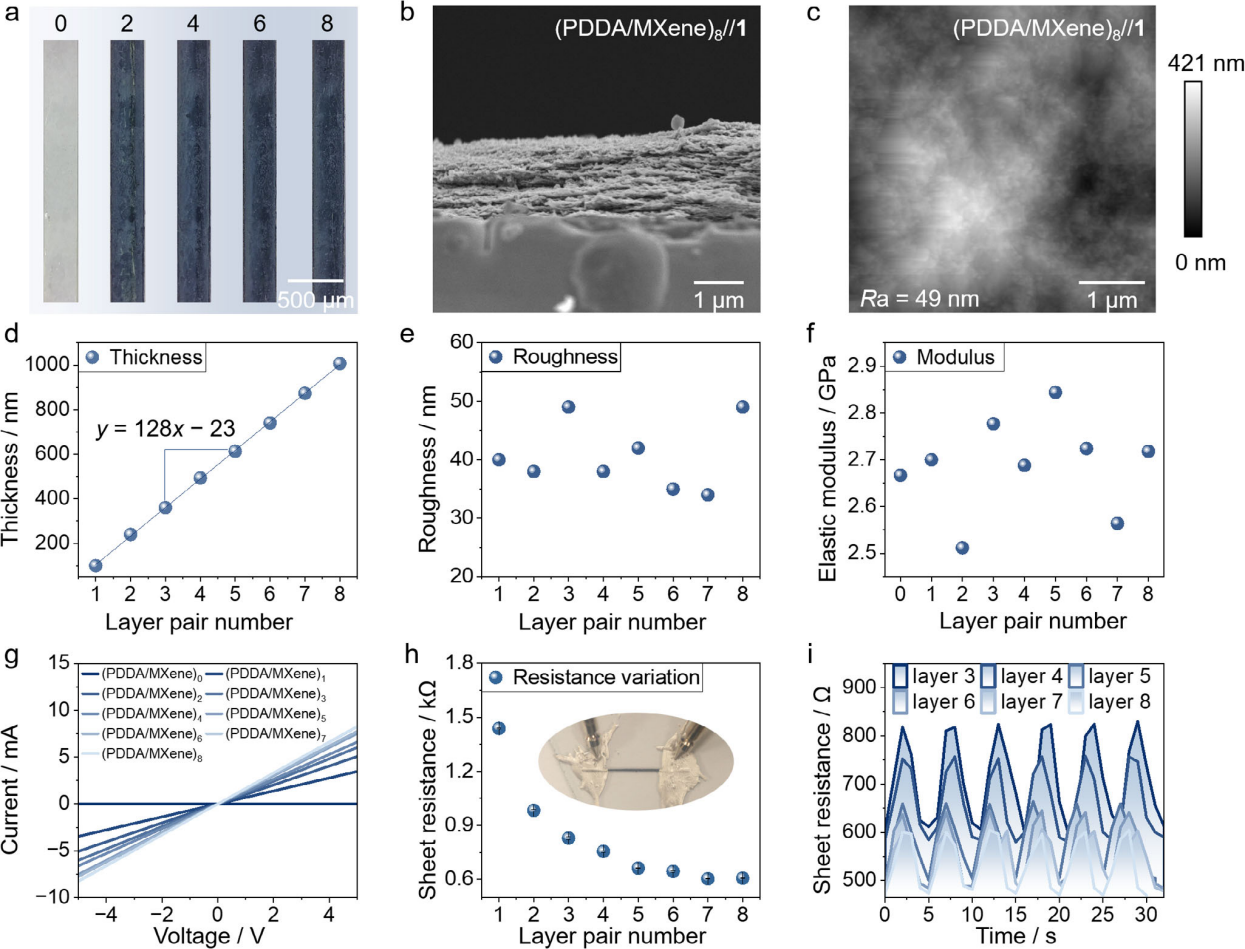
Structural optimization and conductive properties of the composite material P^2^//**1**. (a) Optical photographs of composites having different number of MXene layers (0–8 layers) on the surface of (PDDA/PSS)_5_//**1**. (b) SEM image of the surface of (PDDA/MXene)_8_//**1**. (c) AFM image of the surface of (PDDA/MXene)_8_//**1**. (d) Relationship between the number of MXene layers and the thickness of the composite material. (e) Surface roughness (as measured by the AFM) of P^2^//**1** for composites having different number of MXene layers. (f) Mechanical characterization, via the Young's modulus (determined by using a universal testing machine) of the composites P^2^//**1** having different numbers of MXene layers. (g) *I–* *V* curves of P^2^//**1** with different number of MXene layers. (h) Dependence of the electrical resistance of P^2^//**1** on the number of MXene layers (*n* = 3). (i) Variation in the electrical resistances of the composite structures having different number of MXene layers during periodic deformation (bending).

Control over the compactness and uniformity of the MXene layers in a composite material—determining both the electrical conductivity and retention of structural integrity during deformation—is essential to optimize its sensing performance (Figure 2). Figure S7 illustrates the expected effect of bending on conductivity, where the layer of MXene nanosheets establishes a conductive network on the surface. When the hybrid material bends, the MXene layer is expected to undergo microscopic deformation, causing changes in electrical resistance. With the MXene being the conductive component, this also implies that the conductivity is determined by the thickness of the MXene‐PDDA layer. To verify this hypothesis, composites with varying number of MXene layers (1–8) were prepared. Due to the strong visible absorption of the MXene layer as apparent from its dark color, increasing the number of layers also increases the shade of the color of the composite crystals from transparent to deep black (Figure 2a). For the thickest material in the series, (PDDA/MXene)_8_//**1**, SEM images of the cross‐section indicate that the MXene layers adhere firmly to the substrate (Figure 2b and Figure S8). The thickness increases linearly with the number of layers (*R* ^2^> 0.99), reaching approximately 1 µm at 8 layers, with an average thickness of about 125 nm per MXene‐PDDA layer (Figure 2d). To explore whether thickness variation impacts the samples, additional SEM measurements were performed on three different (PDDA/MXene)_8_//**1** samples, and the results showed that the thickness variations were minimal (Figure S9). Surface roughness analysis based on AFM profiles showed that the surface of the MXene composite remains relatively smooth (*R*_a_ ≈ 30–50 nm), and the roughness does not change significantly with the number of layers (Figure 2c,e and Figure S10).

The relatively dense packing of the MXene sheets, which naturally tend to stack with each other due to their flat shape, is beneficial for forming uniform layer. In Figure 2f, mechanical performance tests demonstrated that P^2^//**1** with different number of MXene layers maintained excellent flexibility (Figure S11), while the *I*–*V* profile confirmed their good conductivity (Figure 2g). Resistance measurement results showed that as the number of MXene layers increased, the resistance of the composite crystals decreased from 1.44 kΩ (1 layer) to about 0.61 kΩ (8 layers) with a clear negative exponential relationship (Figure 2h). We were able to discern two regimes: for 1–5 layers (from 1.44 kΩ to 0.67 kΩ), the resistance decreases significantly (about 56%) with increasing layers, reflecting the additive effect of thickness on the conductivity. Above about 5 layers, however, the resistance plateaus, indicating that the contribution from the additional layers is marginal. As shown in Figure 2i, the resistance of P^2^//**1** with different number of MXene layers responded by periodic variations to reversible bending. Considering the conductivity, materials utilization, complexity of preparation, and cost, we suggest that the structure having 5 layers of MXenes is the optimal choice from the performance perspective. This specific composition responds with good balance between the resistance and materials cost, ensuring a sufficiently low resistance to meet the sensing requirements while at the same time being accessible via simple preparation process.

### 1.3 Strain Detection and Bending Sensing of the Composite Materials

**FIGURE 3:**
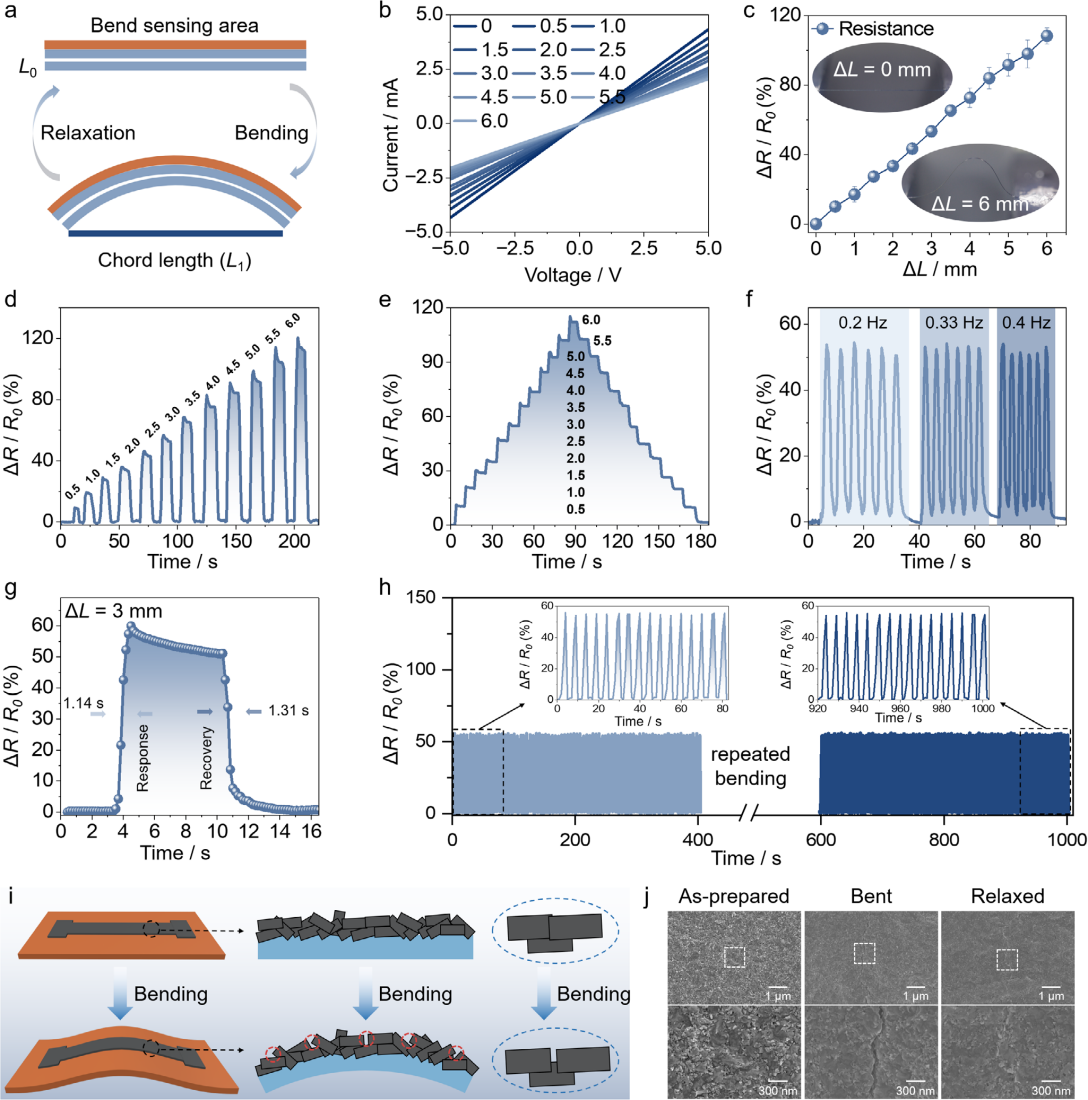
Performance characterization and bending sensing of the composite materials. (a) Schematic diagram defining the length change varied during the bending, where *L*_1_ is the length after change, *L*_0_ is the initial length, and Δ*L* = *L*_0_–*L*_1_. (b) *I–* *V* curves of P^2^//**1** at different chord length changes Δ*L* = 0–6 mm. (c) Relationship between the resistance and the bending expressed by the change in chord length Δ*L* = 0–6 mm, where Δ*R* = *R*_1_–*R*_0_. (d) Time‐dependent change in the resistance of P^2^//**1** when it is bent to different chord lengths Δ*L* = 0–6 mm (the deformation bending and recovery process of hybrid crystals is about 1–4 s). (e) Time‐dependent change in resistance during bending and unbending expressed as different chord lengths Δ*L* = 0–6 mm (the deformation bending and recovery process of hybrid crystals is about 1–2 s). (f) Time‐dependent resistance changes of P^2^//**1** at different bending frequencies and chord length change Δ*L* = 3 mm (the deformation bending and recovery process of hybrid crystals is about 1–3 s). (g) Response time analysis of P^2^//**1** at bending frequency of 0.4 Hz and chord length change Δ*L* = 3 mm. (h) Time‐dependent resistance changes of P^2^//**1** at bending frequency of 0.2 Hz. (i) Schematic diagram of the microscopic mechanism for increased resistance during composite material bending. (j) SEM images of P^2^//**1** in different states showing the evolution of surface microcracks. During the preparation of the hybrid crystal, the MXene sheets pack tightly, and form a conductive network. When the material is bent, microcracks and gaps appear on the surface of the MXene coating. Upon relaxation of the external force, these microcracks partially close.

Deformation sensitivity is the key performance indicator for flexible strain sensors. To study the effect of bending deformation on the electrical properties of the composite material, we designed a bending device whose deformation is controlled by varying the distance between the two ends of the hybrid crystal, defined as Δ*L* = *L*_0_–*L*_1_, where *L* is the distance between the two termini of a hybrid crystal after and before bending (chord length), while the resistance was simultaneously monitored (Figure 3a and Figure S12). As shown in Figure 3b, all *I*–*V* curves recorded at different bending degrees (Δ*L* = 0–6 mm) exhibit linear ohmic characteristics, with the slope decreasing with increasing bending, corresponding to increasing resistance. To exclude the influence of the contact points on the electrical response of the material, a control experiment was carried out. As shown in Figure S13, the probes were placed at the front and back ends of the conductive silver‐paste electrodes, respectively. The resulting *I*–*V* curves were nearly identical, indicating that the contact effect was small. Quantitative analysis revealed an approximately linear relationship between the relative resistance change (Δ*R*/*R*_0_) and bending deformation (Δ*L*), with sensitivity *S* = d(Δ*R*/*R*_0_)/d(Δ*L*) of about 0.18 mm^−1^ (Figure 3c and Figure S14). Similar linear relationships were also observed for three independently prepared samples, further confirming the reproducibility of the bending‐response behavior. Figure 3d,e shows the time‐dependent measurements of the response of P^2^//**1** with bending. When the material was bent from a straight state (Δ*L* = 0 mm) to different degrees (Δ*L* = 0.5–6.0 mm) in 0.5 mm increments, the electrical resistance rapidly increased to a stable value. When the external force was released and the original state was restored, the resistance decreased.

To evaluate the material's performance under periodic deformation conditions, the response characteristics at different bending frequencies (0.2–0.4 Hz) were tested (Figure 3f). The results showed that P^2^//**1** exhibited stable and consistent resistance responses at all frequencies, with signal waveforms synchronized with the bending‐release cycle, without obvious lag or attenuation. Through time‐resolved measurements of a single motion cycle of the composite material (0.2–0.4 Hz), the material's response time was determined to be approximately 1.14 s, and the recovery time was approximately 1.31 s (Figure 3g and Figure S15). In Figure 3h, durability testing in cyclic operation mode showed that P^2^//**1** maintains a stable electrical response after 1000 s of bending (Δ*L* = 3 mm), with almost no change in response rate, demonstrating excellent mechanical durability. The effects of bending on the material were further inspected by SEM (Figure 3i,j). Before bending, the MXene layer surface appeared relatively compact and smooth, with nanosheets tightly stacked to form a continuous conductive network. Upon bending, microcracks and gaps appeared on its surface that were mainly distributed in the areas of increased strain. These microstructural changes significantly affect the electron transport paths: cracks and gaps force electrons to travel over longer distances or through fewer contact points, thereby increasing the overall resistance of the material. Notably, the sizes of these microcracks (typically of sizes that range from tens to hundreds of nanometers) were insufficient to disrupt the conductive film, so the material maintained relatively good conductivity in the bent state, albeit with increased resistance. When the external force was released, the microcracks partially closed, however the surface did not completely return to its original state, leading to reorganization of the conductive layer, which can be related to the changes in resistance observed during reversible bending [54]. Due to the random nature of crack formation on the surface of the composite materials, it is difficult to establish a quantitative relationship between crack size or number and the electrical resistance (Figure S16).

### 1.4 Wide‐Temperature‐Range Conductivity and Low‐Temperature Sensing Capability

**FIGURE 4:**
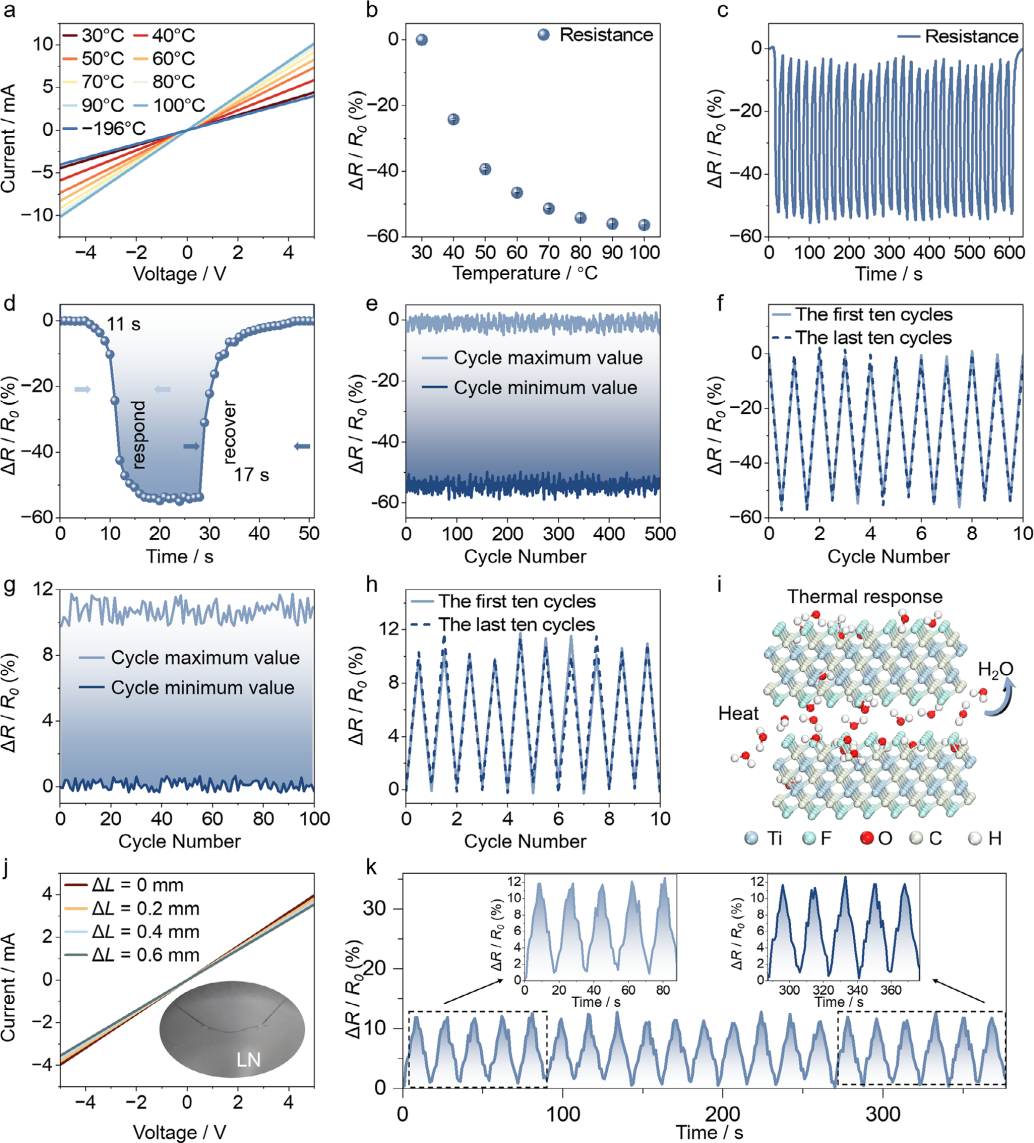
Temperature‐dependent electrical properties and low‐temperature sensing capability of the composite materials. (a) *I*–*V* curves of P^2^//**1** at different temperatures. (b) Dependence of the resistance of P^2^//**1** on the temperature in the 30°C–100°C range (*n* = 2). (c) Time‐dependent resistance changes of P^2^//**1** during cycling between 30°C and 100°C. (d) Response time analysis of P^2^//**1** resistance during switching between 30°C and 100°C. (e) Resistance stability test of P^2^//**1** after 500 cycles between 30°C and 100°C. (f) Comparison of the resistance changes between the first ten cycles and last ten cycles (from the 500 cycles shown in panel e) of P^2^//**1** cycling between 30°C and 100°C. (g) Resistance stability test of P^2^//**1** after 100 cycles between 30°C and −196°C. (h) Comparison of resistance changes between the first 10 cycles and last 10 cycles (from the 100 cycles shown in panel g) of P^2^//**1** cycling between 30°C and −196°C. (i) Schematic diagram of the microscopic mechanism for resistance decrease with increasing temperature, explaining the optimization of conductive networks due to removal of adsorbed water molecules. (j) *I*–*V* curves of P^2^//**3** at −196°C under different bending degrees (Δ*L* = 0.0–0.6 mm). (k) Time‐dependent resistance changes of P^2^//**3** during bending (Δ*L* = 0.6 mm) at −196°C, demonstrating dynamic response capability in low‐temperature environments.

The effect of temperature on the electrical properties of a given material is one of the key indicators for evaluation of its environmental adaptability, particularly in view of its applications in sensors that operate at nonambient temperatures. We therefore studied the conducting properties of P^2^//**1** at various temperatures in the range from −196°C to 100°C (Figure 4a). The *I–* *V* measurements confirmed the favorable conductivity of P^2^//**1** at low temperature (−196°C) and close to ambient temperature (30°C), as well at temperatures above the ambient (100°C). All *I*–*V* curves showed linear ohmic trend that confirm stable electrical transport capability over a wide temperature range (at least, for an organic crystalline material). To study in detail the temperature effect on conductivity, the resistance of P^2^//**1** in the range from 30°C to 100°C was also recorded (Figure 4b). The results showed that, over the temperature range of 30°C–100°C, the relative resistance change (Δ*R*/*R*_0_) of the composite material followed an approximately exponential decreasing trend, reaching about −60% at 100°C (i.e., the resistance decreased to 40% of its initial value). Because the temperature dependence is nonlinear over this range, a single constant temperature coefficient is not sufficient to describe the full response. Similar temperature‐dependent trends were observed for multiple independently prepared samples, further supporting the reproducibility and generality of the thermal response. In Figure 4c,d, the time‐resolved measurements show resistance changes of P^2^//**1** over time when cycled between 30°C and 100°C with a response time of about 11 s and a recovery time of about 17 s.

The material stability under fluctuating temperatures is crucial for practical applications in variable‐temperature environments. To that end, P^2^//**1** was subjected to thermal cycling between different temperatures and its resistance was monitored. In an experiment that spanned 500 cycles between 30°C and 100°C (Figure 4c–f), P^2^//**1** exhibited excellent thermal stability: during each thermal cycle, the resistance switched between low and high temperatures without obvious drift or attenuation (Figure 4f). The resistance‐temperature curves of the first ten cycles and the last ten cycles were almost identical, and thus the material demonstrated good performance after long‐term thermal cycling. The low‐temperature thermal cycling tests (30°C to −196°C) equally confirmed the high temperature stability (Figure 4g,h). However, due to the hardening of the conductive silver paste at low temperatures, the adhesive strength deteriorates over multiple cycles, which makes the durability characterization possible only for fewer cycles. The negative temperature coefficient resistance (Figure 4b–f) can be attributed to the conduction mechanism of the MXene layer (Figure 4i). At room temperature, water molecules are adsorbed on the surface between the MXene layers that increase the distance between nanosheets and partially obstruct the electron transport. As the temperature increases, the water molecules are gradually removed, the average interlayer distance decreases, nanosheets arrange to pack more tightly, and this reduces the overall resistance [55, 56]. At low temperatures, the conductivity slightly increases compared to room temperature, an effect that is attributed to the hopping conduction mechanism, where charge carriers move between localized states due to interlayer contact and structural disorder [57, 58]. To further demonstrate the role of water molecules, a “breathing experiment” on the composite material (relative humidity = 30%) was performed. It was observed that as the water molecules from human breath increased, the resistance of the material increased. Upon stopping the breath, the water molecules evaporated, and the resistance decreased (Figure S17). However, perhaps the most striking result is our observation that P^2^//**3** maintains sensitivity to mechanical deformation even at low temperatures (−196°C) (Figure 4j,k). *I–* *V* measurements at liquid nitrogen temperature (Figure 4j) showed that as the bending increases (Δ*L* = 0−0.6 mm) the resistance simultaneously increases, and it exhibits strain sensitivity characteristics similar to those observed at room temperature. The time‐resolved measurements in Figure 4k further confirmed that at −196°C, the material exhibits rapid and reversible responses to bending. The hardening of the silver paste leads to a decrease in adhesive strength, which results in a smaller deformation range during low‐temperature testing. In the cryogenic tests, conductive silver paste was used as the adhesive electrical contact, and its reduced adhesion at low temperature may introduce additional uncertainty into the cycling data; therefore, these results mainly demonstrate the feasibility of cryogenic sensing rather than strict long‐term cycling durability. This ability of the material to not only deform but to also maintain the deformation sensitivity at low temperature is relevant as it is poised to provide a unique opportunity for sensor development in challenging applications such as space exploration and low‐temperature research. Table S5 summarizes the operating temperature ranges of this study and some previously reported MXene‐based and crack‐based flexible sensors. The results show that there are differences in the operating temperature ranges of the devices, and the composite material in this study operates stably in the range from −196°C to 100°C [59, 60, 61, 62].

### 1.5 Application to Integrated Sensing Systems

**FIGURE 5:**
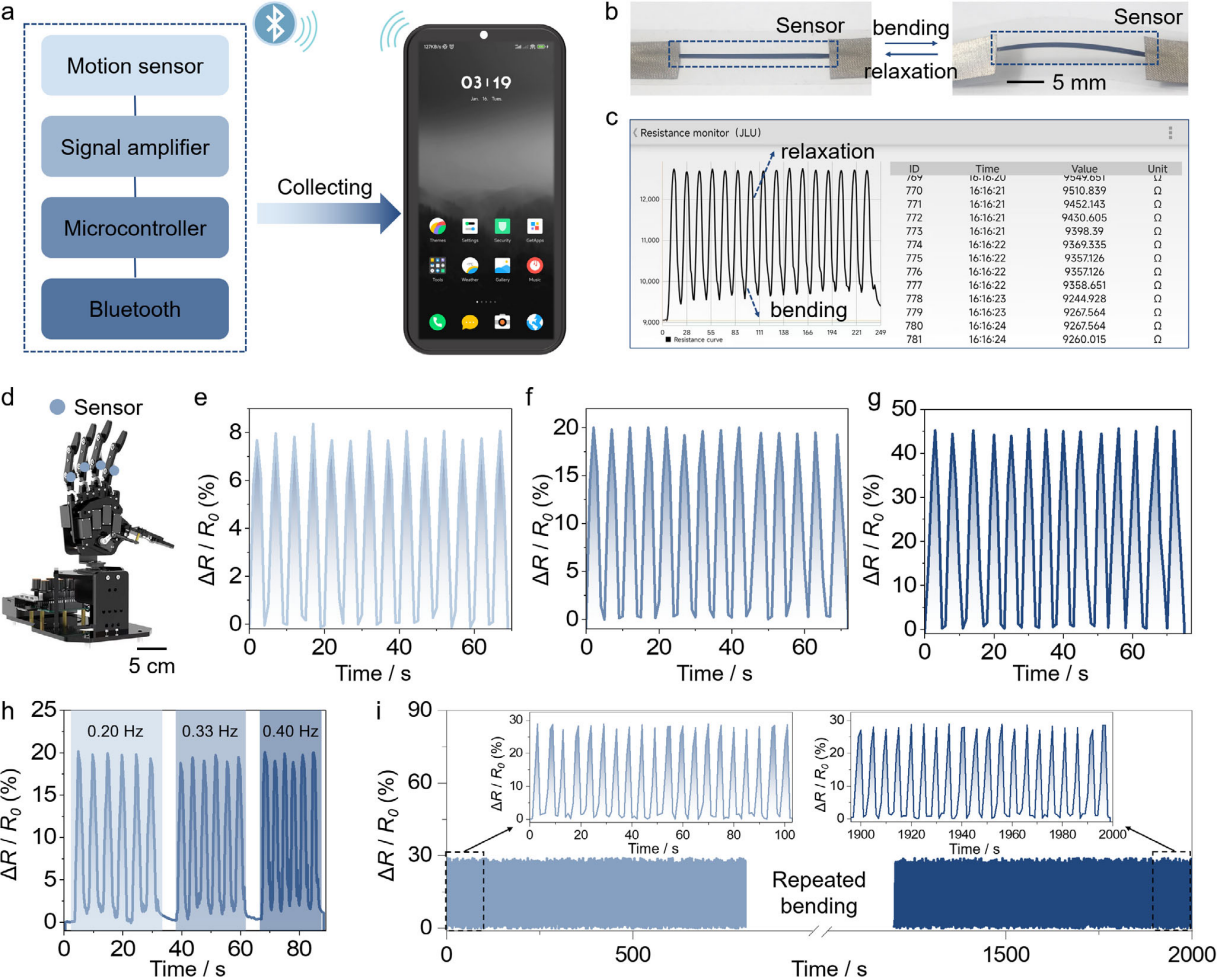
Integrated sensing system and proof‐of‐concept application of the composite material. (a) Structural schematic diagram of the flexible sensor system based on an organic crystal‐MXene composite material, showing the integration of a microcontroller, a signal processing circuit, and a Bluetooth communication module. (b) Photographs of real‐time response of P^2^//**1** crystal sensor during repeated bending process. (c) Smartphone interface display showing real‐time monitoring of P^2^//**1** sensor resistance change, demonstrating the system's wireless data transmission and visualization capabilities. (d) Images of the composite material as a sensor for monitoring mechanical hand movements. (e–g) Resistance vs time curves of the sensor at different degrees of mechanical hand bending, showing linear response characteristics with increasing resistance as bending degree increases where e, f, and g correspond to 1, 2, and 3 in Figure S19, respectively. (h) Resistance vs time changes of the sensor at different mechanical hand bending frequencies. (i) Cyclic response curves of sensor resistance vs time during repeated bending of the mechanical hand.

To demonstrate the potential of the composite materials for practical applications, we designed an intelligent flexible sensing system (Figure 5a and Figure S18). The sensing element, made of P^2^//**1**, is connected to a microcontroller using silver conductive adhesive in a signal acquisition circuit based on the voltage division principle. The system wirelessly transmits data to a smartphone application via a Bluetooth module, enabling real‐time monitoring and data visualization. The system is powered by a 3.3 V regulated power supply and uses capacitive filtering to improve signal quality and to ensure accurate measurements even under dynamic conditions. This integrated design endows the sensing system with portability, capability for real‐time data collection, and wireless communication, rendering it suitable for a variety of mobile monitoring applications (Figure S19). As shown in Figure 5b,c, flexible sensing tests confirmed that P^2^//**1** produces stable, repeatable resistance signals during periodic bending‐and‐recovery processes. The smartphone interface displays the resistance changes in real time and concomitantly with the periodic deformation: the resistance rapidly increases during bending (marked as “bending” areas in the figure), and rapidly decreases during recovery (marked as “relaxation” areas in the figure), with no obvious lag or signal instability throughout the process (Figure 5c and Movie S1).

To further evaluate the feasibility in human‐machine interaction applications, we integrated the sensor into a mechanical finger to simulate human hand movement (Figure 5d). The experimental design included various finger action patterns: different degrees of bending (Figure 5e–g and Figure S20), repeated movements at different frequencies (Figure 5h), and long‐term continuous monitoring (Figure 5i). The results showed that the sensing system can accurately detect and distinguish different degrees of finger bending; as the bending degree increases (from slight to maximum bending), the resistance signal correspondingly strengthens, indicating that the sensor has good resolution across the entire motion range (Figure 5e–g and Figure S21 and Movie S2). In dynamic tests, the sensor exhibited consistent and fatigueless response at different frequencies (0.2–0.4 Hz) of finger movement, with the signal waveforms clearly reflecting the movement rhythm and without obvious frequency dependence or signal distortion (Figure 5h). Continuous monitoring results showed that the sensor maintained stable performance after up to 2000 s of continuous operation, demonstrating the system's reliability in practical applications (Figure 5i). We also evaluated the monitoring performance of different fingers. In Figure S22, when sensors were installed separately on the index finger and the ring finger, comparative tests showed that the system exhibited stable responses to the bending actions of different fingers; when two sensors were used in a series, the system was able to distinguish between single finger bending and cooperative action of both fingers (Figure S21 and Movie S3), providing possibilities for complex gesture recognition. Durability tests showed that the sensing system maintained stable detection performance after continuous use for one week, demonstrating the long‐term stability of the material and the system in real environments (Figure S22). As shown in Figures S19–S21, multiple independently prepared sensors exhibited consistent bending responses on the robotic finger, further confirming the reproducibility and general applicability of the sensor design. Combined with the excellent performance of the material in low‐temperature conditions, our research implicates that sensor materials based on flexible organic crystal‐MXene composite materials are promising for deformation monitoring in extreme environments, which could be relevant to space exploration.

## 2 Conclusions

In this research, we have developed an approach for preparation of flexible sensing materials based on elastic organic crystal‐MXene composite architecture that are electrically conductive and can be bent with high sensitivity across a wide temperature range from −196°C to 100°C as an orthogonal approach to existing electronic devices for extreme environments. Through a controlled electrostatic layer‐by‐layer self‐assembly protocol, we prepared conductive MXene sheets on the surface of organic crystals that impart electrical conductivity while preserving the inherent flexibility of the crystal. The composite materials maintained excellent conductivity across a wide temperature range, even after repeated bending at various temperatures. They also exhibited high sensitivity and rapid response with linear resistance‐deformation profile. We note that the composite materials maintained responsive capability to mechanical deformations even at liquid nitrogen boiling temperature (−196°C), which is accessible in most chemistry labs. Application demonstrations proved that sensors based on this material could accurately respond to bending motions such as for example those of human bodies, with good response speed, stability, and durability. These innovative materials break through the temperature limitations of traditional flexible electronic materials, providing a new technical pathway for flexible sensing in extreme environments.

## 3 Experimental Section

### 3.1 Materials

The solvents and starting materials used in the syntheses were obtained from commercial sources and used without further purification. The compounds **1**–**3**, namely (*E*)‐2‐(4‐fluorophenyl)‐3‐(naphthalen‐1‐yl)acrylonitrile, (*Z*)‐2‐([1,1′‐biphenyl]‐4‐yl)‐3‐(anthracen‐9‐yl)acrylonitrile, and (*Z*)‐3‐(furan‐2‐yl)‐2‐(4‐(((*E*)‐2‐hydroxy‐5‐methylbenzylidene)amino)phenyl)acrylonitrile, were synthesized following established procedures (Figures S1–S3) [45, 46, 47]. To prepare the samples, dichloromethane solutions of compounds **1**‒**3** were placed in test tubes. Approximately triple volume of ethanol was then added along the walls of the tube without disturbing the surface of the solution and the solutions were left undisturbed for diffusion to occur. Needle‐shaped crystals of the compounds **1**‒**3** were obtained after one to two weeks at room temperature.

### 3.2 Preparation of the Hybrid Crystals

The pristine organic crystals **1**–**3** were immersed in an aqueous solution of poly(diallyldimethylammonium chloride) (PDDA, 1.0 mg mL^−1^) for 20 min to allow sufficient adsorption of the positively charged layer. The crystals were then gently rinsed with distilled water for 1 min to remove excess physically adsorbed species. Subsequently, the crystals were immersed in an aqueous solution of poly(sodium 4‐styrenesulfonate) (PSS, 1.0 mg mL^−1^) for another 20 min, followed by the same rinsing procedure. By repeating the PDDA/PSS deposition cycle five times, polymer‐coated crystals denoted as ((PDDA/PSS)_5_//**1**‒**3**) were obtained. To introduce the conductive layer, the polymer‐coated crystals were first immersed again in the PDDA solution for 20 min to modify the surface charge, followed by rinsing. Subsequently, a MXene aqueous dispersion (10 mg mL^−1^) was uniformly deposited onto one of the two wide surfaces of the crystal using a syringe. After the coating was completely dried, the crystal was re‐immersed in the PDDA solution. This cycle “MXene deposition–drying–PDDA modification” was repeated to yield the conductive and mechanically flexible hybrid composites PDDA/MXene//PDDA/PSS//**1**–**3** (P^2^//**1**‒**3**; Figure 1d).

### 3.3 Preparation of the MXene

To prepare the Ti_3_C_2_T*_x_* suspension, Ti_3_AlC_2_ powder was purchased from Energy Chemical. 2.0 g of the Ti_3_AlC_2_ powder was mixed with 2.0 g LiF and 30 mL 9.0 M HCl, and stirred at 35°C for 24 h. The mixture was washed and separated by centrifugation at 3500 rpm several times until the pH of the supernatant was 7. Then the sediment of MXene was collected and mixed with 40 mL of deionized water. The mixture was sonicated under N_2_ atmosphere for 1 h and then centrifuged at 1370*g* for 1 h to obtain the MXene suspension. The MXene powder was prepared by freeze‐drying of the MXene suspension.

### 3.4 Quantification of Materials Properties

To quantitatively evaluate the performance of the sensor, the following formulas and data fitting procedures were used:

#### 3.4.1 Relative Resistance Change Rate (Δ*R/R*_0_)

The relative resistance change rate was used to measure the electrical signal response, and is defined as Δ*R* = *R*_1_–*R*_0_ where *R*_0_​ is the initial resistance of the crystal in its undeformed state and *R*_1_ is the resistance after deformation or temperature change, so that Δ*R/R*_0_ *=* (*R*_1_–*R*_0_)*/R*_0_.

#### 3.4.2 Displacement (Δ*L*)

In this work, Δ*L* was used to describe the bending deformation because it is a directly controllable experimental parameter and reporting Δ*L* provides a more direct and consistent basis for comparison under the present testing configuration. This approach was used as a measure of strain and is defined as the change in chord length between the two ends of the crystal before and after bending, Δ*L* = *L*_0_–*L*_1_, where *L*_0_​ and *L*_1_​ are the initial and deformed chord lengths, respectively.

#### 3.4.3 Sensitivity (*S*)

The sensitivity is defined as the derivative of relative resistance change rate with respect to displacement and is given by: *S* = d(Δ*R*/*R*_0_)/d(Δ*L*).

#### 3.4.4 Young's Modulus (*E*)

Based on the stress‐strain curve obtained from three‐point bending tests, Young's modulus in the elastic deformation range is calculated using the formula *E* = σ/ϵ, where *σ* is the stress and *ϵ* is the strain. In the present study, *σ* was calculated as 3*PL*/(2*bh* ^2^), and *ϵ* was calculated as 6*Dh*/*L* ^2^, where *P* is the applied load, *L* is the support span, *b* is the sample width, *h* is the sample thickness, and *D* is the displacement.

#### 3.4.5 Data Fitting Procedure

All linear regression analyses in this study (such as the relationship between the MXene coating thickness and number of layers, and the relative resistance vs bending displacement) were performed using the Origin software. The linear slope and the coefficient of determination (*R* ^2^) were extracted using this software to ensure the statistical significance of the data.

### 3.5 Conductivity Testing of the Composite Materials

In this study, conductive silver paste was applied to both ends of the composite crystal samples as electrodes. After allowing the silver paste to dry naturally at room temperature, a stable interface contact was formed with the conductive MXene network. The experiments were conducted using a two‐probe configuration, with high‐precision tungsten needles serving as probes to connect to the silver paste electrodes. These probes were linked to a Keithley 2400 SourceMeter for signal acquisition (Figure S23). The effective distance between the electrodes corresponds to the test length of the sample. Given the excellent conductivity of the silver paste, and the observed good linear characteristics in the *I–V* curves, the influence of contact resistance at the interface was considered negligible in this study.

### 3.6 Experimental Conditions

#### 3.6.1 Standard Three‐Point Bending Test

The standard three‐point bending test was performed using an Instron 5943 Universal Testing Machine to obtain the Young's modulus of the samples. The span for the test was set to 3 mm, with a loading rate of 3 mm min^−1^. Before the test, the width and thickness of the crystal sample were precisely measured using an optical microscope. The stress (*σ*) and strain (*ϵ*) were then calculated using standard material mechanics formulas, and the Young's modulus was determined by fitting the linear region of the stress‐strain curve using the formula: *E* = *σ*/*ϵ*.

#### 3.6.2 Resistance‐Deformation Response and Sensor Performance Characterization

The electrical response of the sensor was assessed using a custom‐designed bending test apparatus (Figure S12). Both ends of the crystal sensor were fixed at the movable end of the rig, and the deformation was precisely controlled by adjusting the distance between the two ends. The deformation was defined as the change in distance, Δ*L* = *L*_0_−*L*_1_, where *L*_0_​ is the initial distance and *L*_1_ is the real‐time distance after bending. The deformation range for Δ*L* was set from 0 to 6 mm, with a bending time to the set distance of approximately 0.5–1.0 s. To further evaluate the dynamic response characteristics of the sensor, the test frequencies were set to 0.20 Hz, 0.33 Hz, and 0.40 Hz. These detailed parameter settings ensure that the sensor exhibits excellent signal stability across different motion frequencies.

#### 3.6.3 Heating/Cooling Method

The heating test (30°C–100°C) was carried out using a heating stage with high‐precision feedback control. For low‐temperature testing (−196°C), the sample was placed in a liquid nitrogen environment. For the temperature measurements, a temperature control and display device (TCS‐300) together with a heating platform was used, and the sample was placed directly on the surface of the heating platform, where the temperature was controlled and monitored by the built‐in feedback system of the stage (Figure S24).

#### 3.6.4 Sensor Placement

In the temperature‐dependent electrical testing, the composite crystal sample was placed in the center of the heating stage to ensure uniform heating. The two ends of the sample were connected using conductive silver paste to lead the electrical signal out of the heating zone to the Keithley 2400 SourceMeter for real‐time monitoring. This setup ensures that the sample responds fully to the temperature changes while preventing the measurement equipment from being exposed to high temperatures.

#### 3.6.5 Atmosphere

All tests were performed in ambient air to study the influence of environmental water on the MXene conductive network.

#### 3.6.6 Ramp Rate and Dwell Times

The heating rate was set to approximately 20°C min^−1^. Once the target temperature was reached, the sample was held at the target temperature for about 2 min to allow thermal equilibrium before recording the data.

### 3.7 Portable Wireless Signal Acquisition System

A portable wireless signal acquisition system was designed in this work for accurate detection and acquisition of the sensing performance of flexible sensors, with the overall architecture of the system illustrated in Figure 5a. Taking the ESP32 module integrated with a Bluetooth communication module and an analog‐to‐digital converter (ADC) as the core control unit, the complete working process of the system is as follows: at the sensor end, the resistance signal of the sensor is linearly converted into a voltage signal via a signal conversion circuit with the configuration of series resistor and an external voltage; the voltage signal is then collected by the ESP32 module, followed by the analog‐to‐digital conversion implemented by its built‐in ADC, as well as the subsequent data analysis and logical processing. The processed sensing data is wirelessly transmitted to the terminal device through the integrated Bluetooth module of the ESP32, and finally the independent‐developed Android application completes the reception, storage, and recording of the data. Thus, a complete closed loop from signal perception, conversion, and processing to terminal data storage is constructed.

## Funding

This work received support from the National Natural Science Foundation of China (Grant Nos. 523B2032, 52373181, 52173164), the Natural Science Foundation of Jilin Province (Grant No. 20230101038JC), the Postdoctoral Fellowship Program of GPSF and China Postdoctoral Science Foundation under Grant No. BX20250094, and Funding from New York University Abu Dhabi (project AD073). Additionally, this material is based on works supported by Tamkeen under the NYUAD RRC Grant No. CG011.

## Conflicts of Interest

The authors declare no conflicts of interest.

## Supporting information




**Supporting File 1**: advs74988‐sup‐0001‐Movie1.mp4.

**Supporting File 2**: advs74988‐sup‐0002‐Movie2.mp4.

**Supporting File 3**: advs74988‐sup‐0003‐Movie3.mp4.

**Supporting File 4**: advs74988‐sup‐0004‐SuppMat.docx.

## Data Availability

The data that support the findings of this study are available from the corresponding author upon reasonable request.
