# Supplementary material for: Organic Crystal‐MXene Composites as Temperature‐Tolerant Strain Sensors
Source: Adv Sci (Weinh). 2026 Mar 30;13(32):e22007. doi: 10.1002/advs.202522007 (PMC13252632; doi:10.1002/advs.202522007)
Supplement: Supplementary file 4 — Supporting File 4: advs74988‐sup‐0004‐SuppMat.docx. [file ADVS-13-e22007-s002.docx]

Supporting Information

**Organic Crystal-MXene Composites as Temperature-Tolerant Strain Sensors**

*Xuesong Yang, Lijie Wang*, Shun Li, Yuhan Zhang, Yongqiang Xu, Haixia Mei*, Liang Li, Ying Tao*, Quan-Hong Yang, Panče Naumov*, Hongyu Zhang**

**Supporting Methods**

**General information**

All solvents and starting materials for syntheses were purchased from commercial sources and were used as received without further purification. Poly(diallyldimethylammonium chloride) (PDDA, Mw. 200,000–350,000), poly(sodium styrene sulfonate) (PSS, Mw. 70,000), PDDA and PSS aqueous solutions were at a concentration of 1.0 mg/mL. Scanning electron microscopy (SEM) images were obtained on the FEI Quanta 450 operated at 5‒10 kV. The emission spectra were recorded on a Maya2000 Pro CCD spectrometer. In order to test their optical waveguiding capability, the crystals were irradiated by the third harmonic (355 nm) of a Nd:YAG (yttrium-aluminum-garnet) laser at a repetition rate of 10 Hz and a pulse duration of about 10 ns. The energy of laser was adjusted by using the calibrated neutral density filters. The beam was focused on a stripe whose shape was adjusted to 3.3 × 0.6 mm by using a cylindrical lens and a slit. The edge emission spectra were recorded on a Maya2000 Pro CCD spectrometer. Atomic force microscopy (AFM) images and surface roughness data were collected using a Bruker Icon-XR AFM in tapping mode. The Young's modulus and stress–strain curves of the crystals were measured using an Instron 5943 Universal Testing Machine employing a three-point bending method. For electrical characterization, *I-V* characteristics and resistance measurements were performed using a Keithley 2400 SourceMeter and a multimeter. A wireless sensing system was developed using an ESP32 microcontroller integrated with a Bluetooth module, designed for data acquisition based on the voltage division principle and powered by a 3.3V regulated power supply. Temperature control was achieved using liquid nitrogen for low-temperature cooling (down to –196°C) and a precision heating stage with high-accuracy feedback control for temperature regulation.

**Supporting Figures**


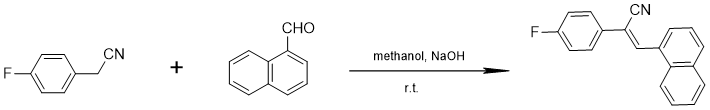


**Figure S1. The synthetic procedure of compound (*E*)-2-(4-fluorophenyl)-3-(naphthalen-1-yl)acrylonitrile (for convenience, hereafter named to as 1).** 4-Fluorophenylacetonitrile (1.36 g, 10 mmol) and 1-naphthaldehyde (1.56 g, 10 mmol) were added to methanol (50 mL). NaOH (0.40 g, 10 mmol) was added and the mixture was stirred for 1 h at room temperature. The reaction mixture was filtered to give a green solid, which was dissolved in dichloromethane and washed with brine. After drying over Na_2_SO_4_, the solvent was removed by vacuum spin evaporation. The resulting crude product was purified by column chromatography using dichloromethane as eluent to obtain compound **1** (2.25 g, 77%) as a green powder.


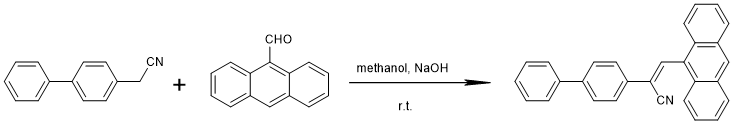


**Figure S2 The synthetic procedure for compound (*Z*)-2-([1,1′-biphenyl]-4-yl)-3-(anthracen-9-yl)acrylonitrile (for convenience, hereafter named to as 2.** 4-Biphenylacetonitrile (1.93, 10 mmol) and 9-anthraldehyde (2.06 g, 10 mmol) were added to ethanol (50 mL). NaOH (0.40 g, 10 mmol) was then added, and the mixture was stirred for 4 h at room temperature. The mixture was filtered to have a yellow solid, which was dissolved in dichloromethane and washed with brine. After drying over Na_2_SO_4_, the solvent was removed by vacuum roto-evaporation. The resulting crude product was purified by column chromatography using dichloromethane as an eluent to obtain compound **2** (3.41 g, 85%) as a yellow powder.


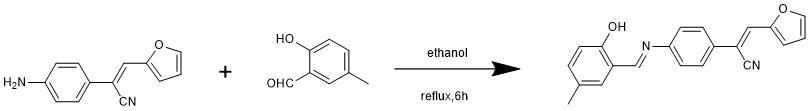


**Figure S3 The synthetic procedure of compound (*Z*)-3-(furan-2-yl)-2-(4-(((*E*)-2-hydroxy-5-methylbenzylidene)amino)phenyl)acrylonitrile (for convenience, hereafter named to as 3.** 2-(4-aminophenyl)-3-(furan-2-yl)acrylonitrile (2.10 g, 10 mmol) and 2-hydroxy-5-methylbenzaldehyde (1.36 g, 10 mmol) were dissolved in ethanol (50 mL). After refluxing for 6 h, the resultant mixture was cooled down to room temperature, and was filtered and washed with ethanol. The crude product was purified by column chromatography using dichloromethane and petroleum ether (V/V= 4:1) as the eluent to produce compound **3** as an orange-red solid (2.71 g, 78% yield).

**
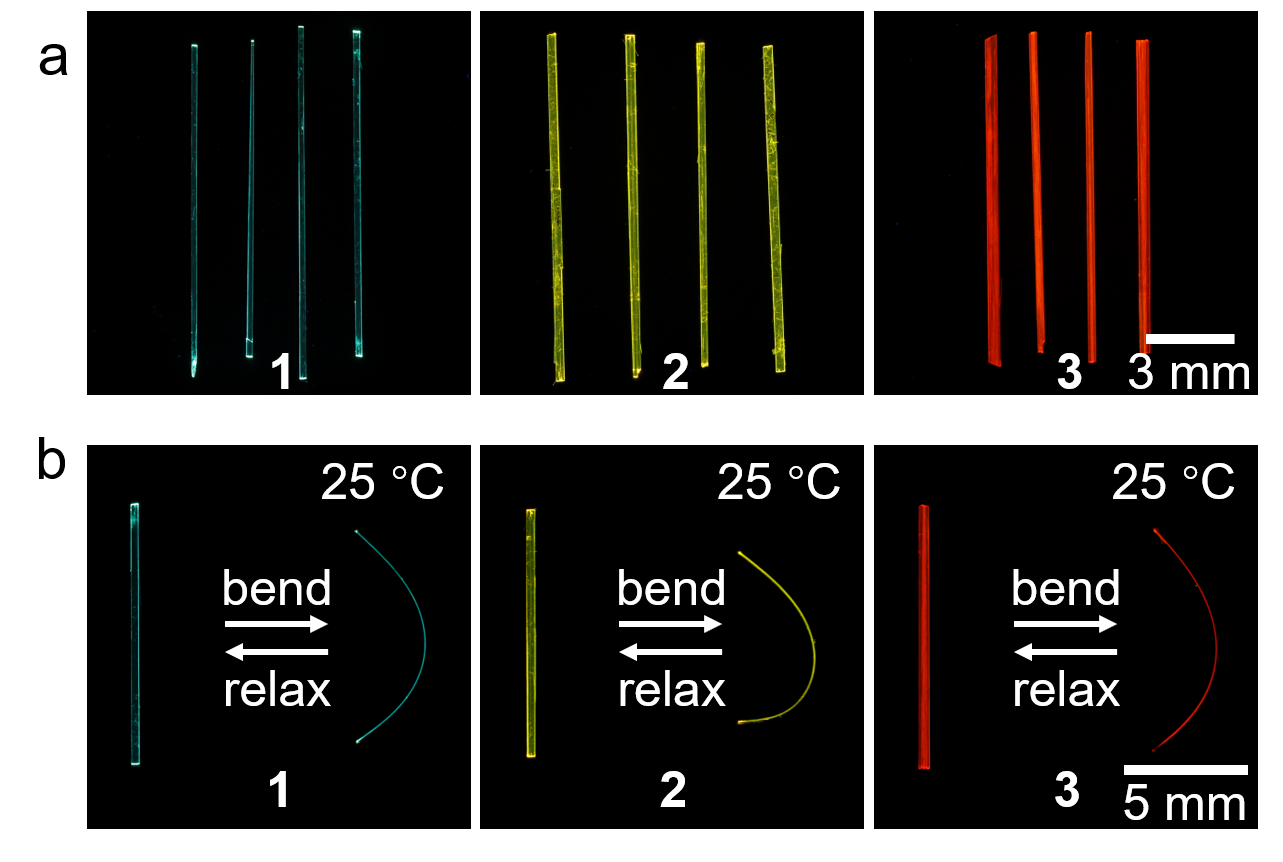
**

**Figure S4. Photographs of the crystal.** (a) Photographs of crystals **1**–**3** taken under UV light (365 nm). (b) Photographs of crystals **1**–**3** bent reversibly at room temperature (25 °C).


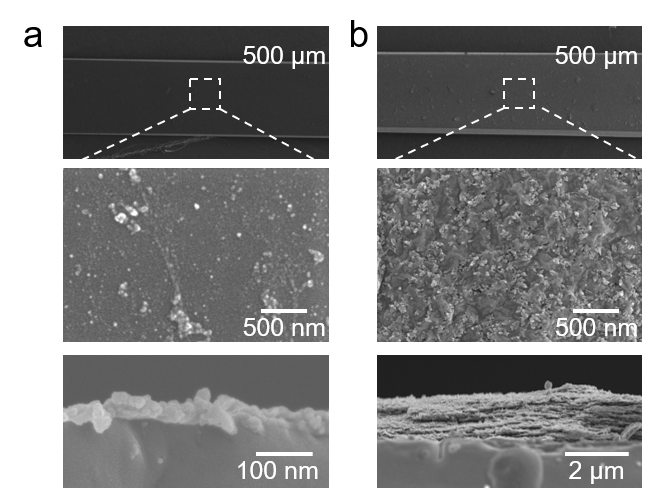


**Figure S5. Scanning electron microscopic images.** (a,b) SEM images of the surface of (PDDA/PSS)_5_//**1** (a) and P^2^//**1** (b).


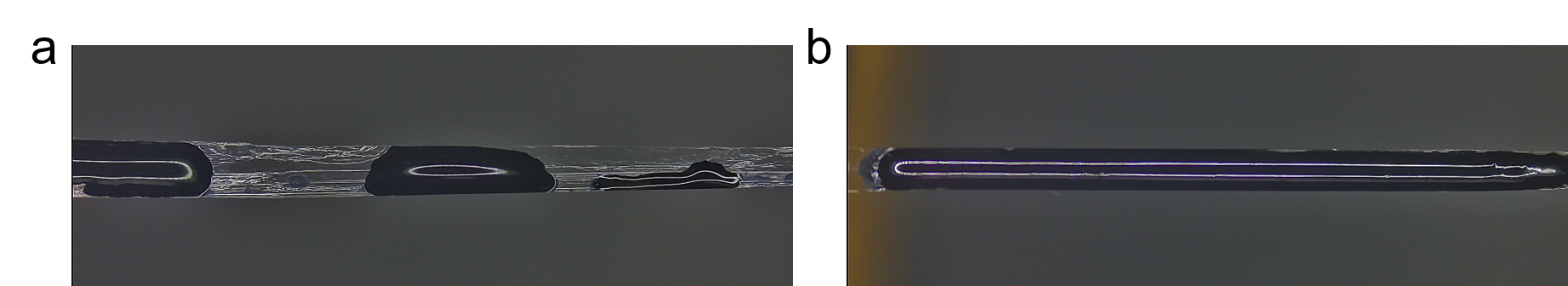


**Figure S6.** **Optical images of the organic crystal before and after surface modification.** (a) The surface of the pristine crystal **1** shows uneven MXene distribution. (b) A uniformly continuous MXene coating on the surface of (PDDA/PSS)_5_//**1.**


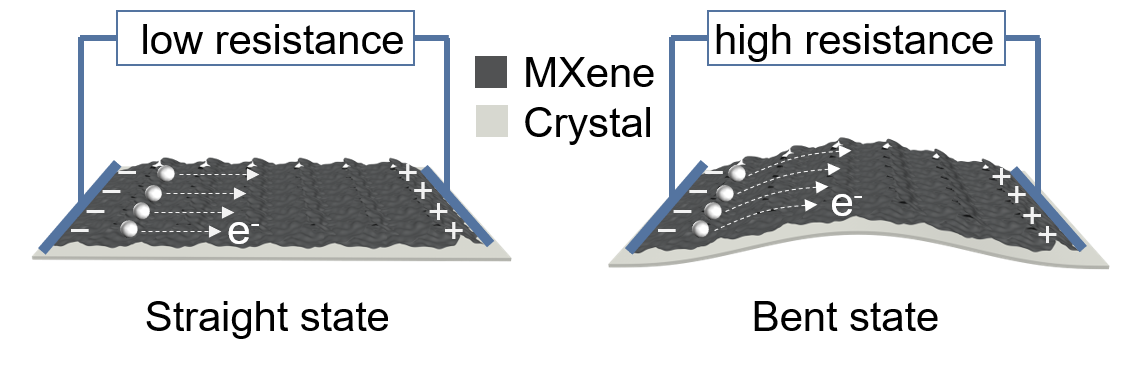


**Figure S7. Schematic diagram of conductivity in the straight and bent state of the hybrid crystals.** The diagram shows mechanism of conductivity modulation in straight and bent states of the material and highlights the role of the MXene layer on its surface in the carrier transport.


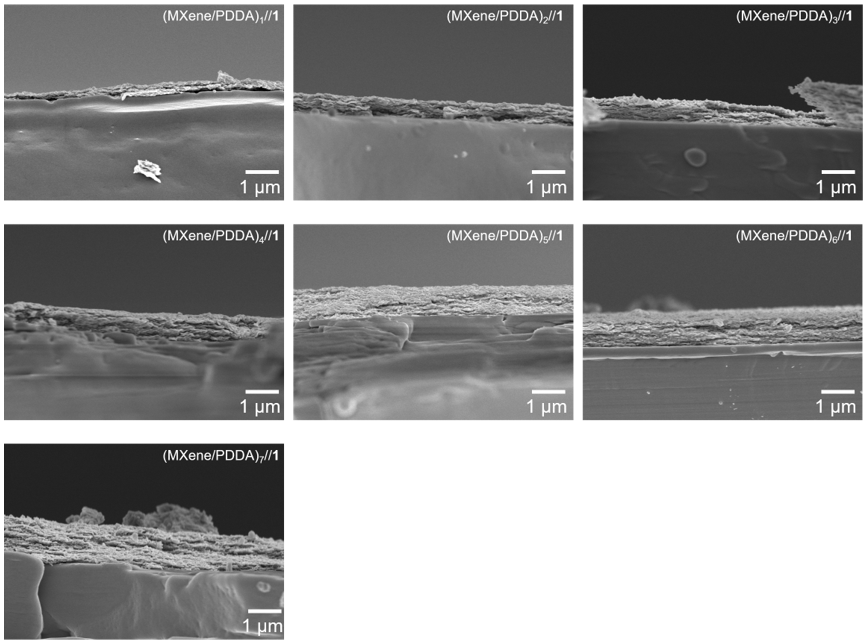


**Figure S8. Photographs of the crystal taken under scanning electron microscope.** The images show cross-sections of hybrid crystals having different number of MXene layers.


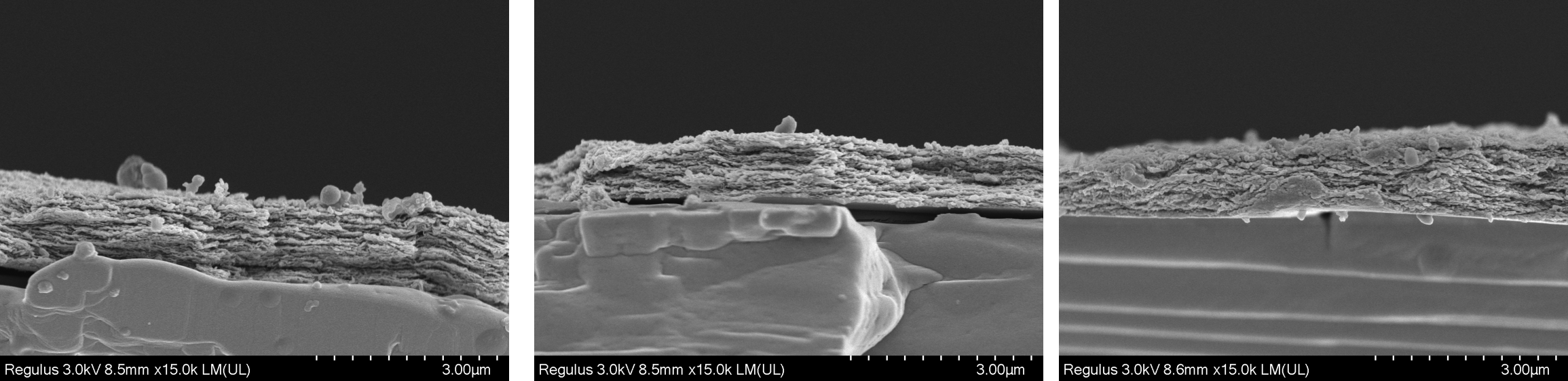


**Figure S9. SEM images.** SEM images of the (PDDA/MXene)_8_//**1** sample.


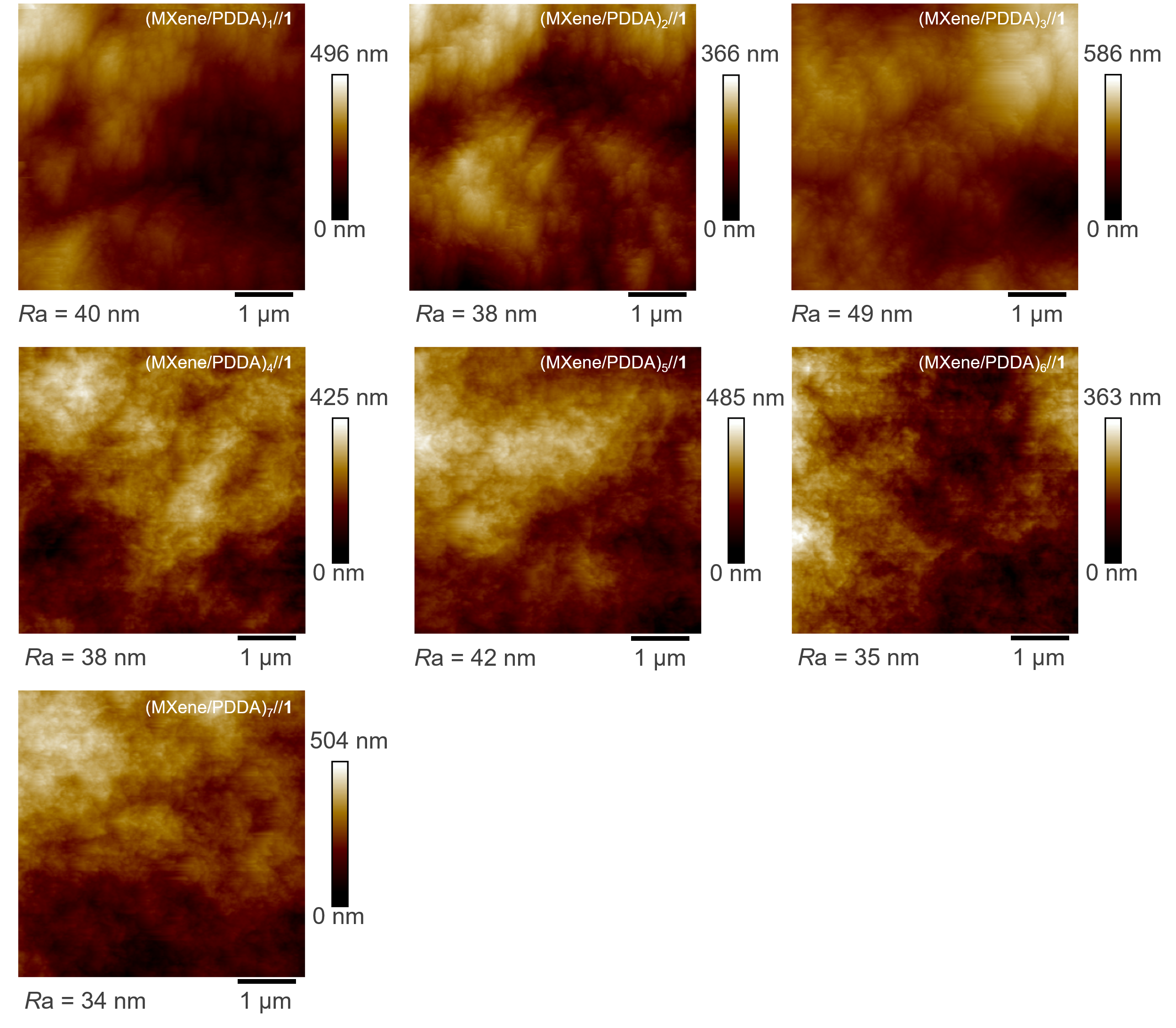


**Figure S10. Surface topography inspected by atomic force microscopy (AFM).** Atomic force microscopy (AFM) images of of P^2^//**1**.


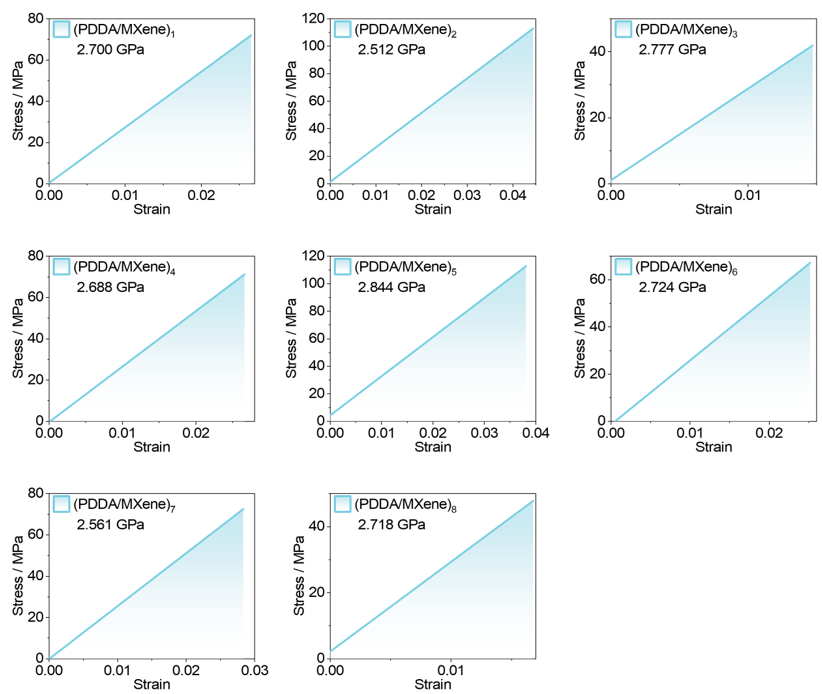


**Figure S11. Three-point bending tests of P^2^//1** **in the linear regime.** The plots show stress-strain curves with different number of MXene layers. Then numbers are the estimated Young’smoduli from each curve.


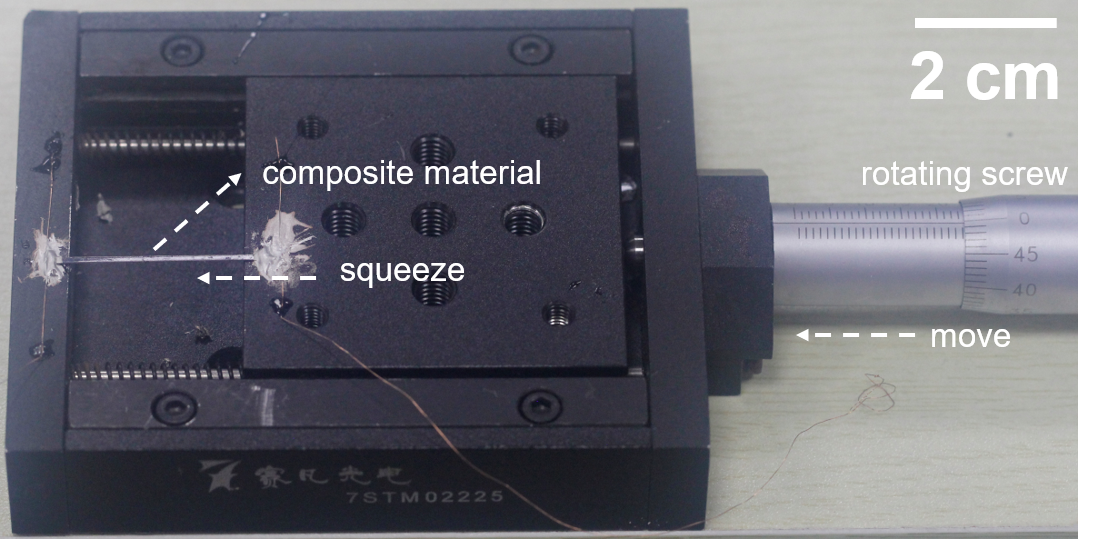


**New Figure S12. Dimensioned photograph of the bending rig used for the electromechanical measurements.** The composite crystal is fixed in the middle of the device, and a 2 cm scale bar is included to indicate the dimensions of the setup.

**
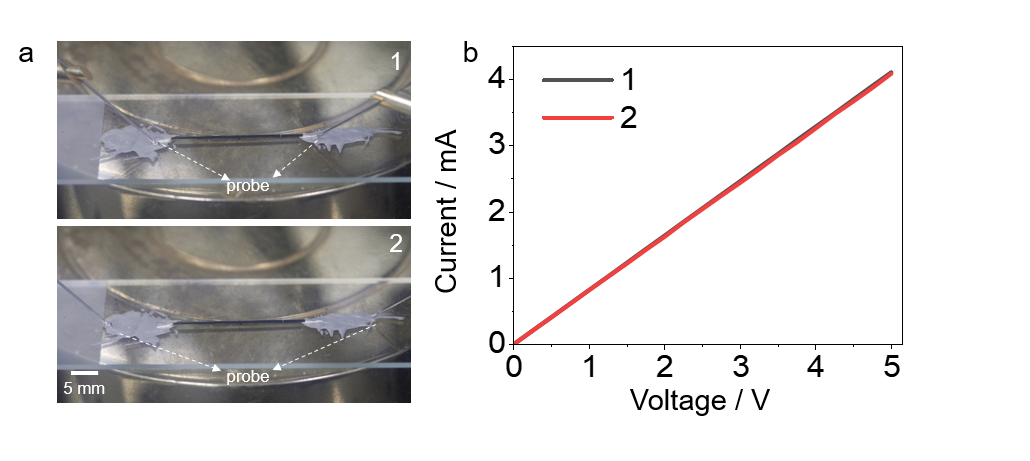
**

**Figure S13. Probe placement at different electrode positions and the corresponding I–V characteristics.** (a) Photographs showing the probe placed at different electrode positions. (b) Comparison of the I–V curves measured at different probe positions.


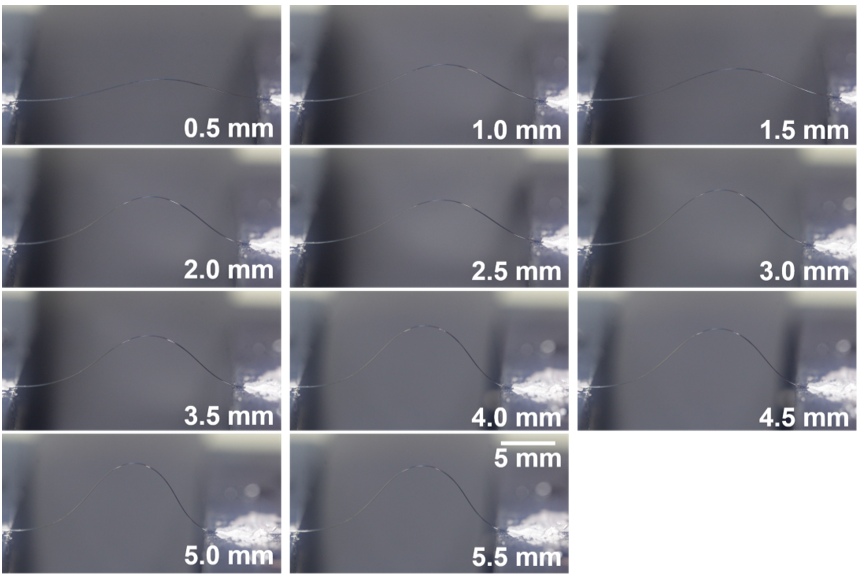


**Figure S14. Photographs of hybrid organic crystals bent.** Photographs of crystals being gradually bent by reducing the distance between their ends.


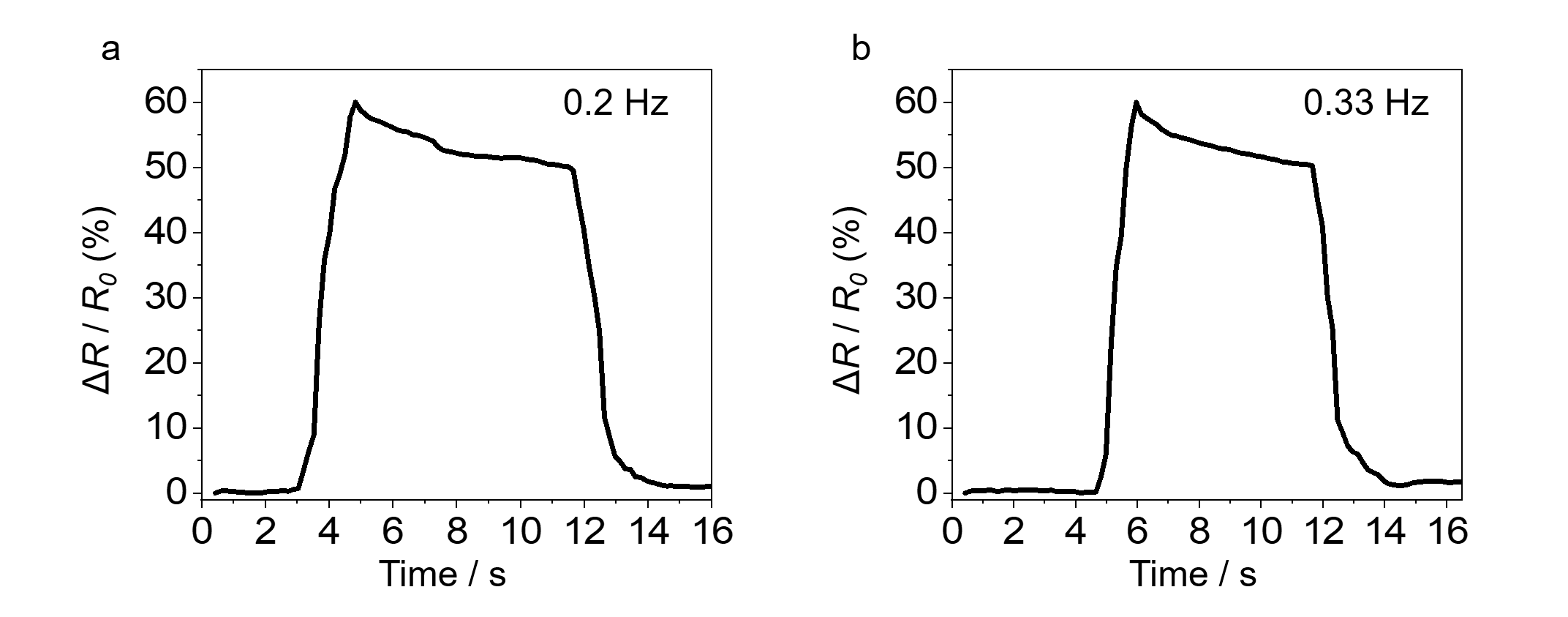


**Figure S15. Variation of the electrical resistance with time.** (a) Response with time of P^2^//**1** at 0.2 Hz bending frequency (length change Δ*L* = 3 mm). (b) Response with time of P^2^//**1** at 0.33 Hz bending frequency (length change Δ*L* = 3 mm).


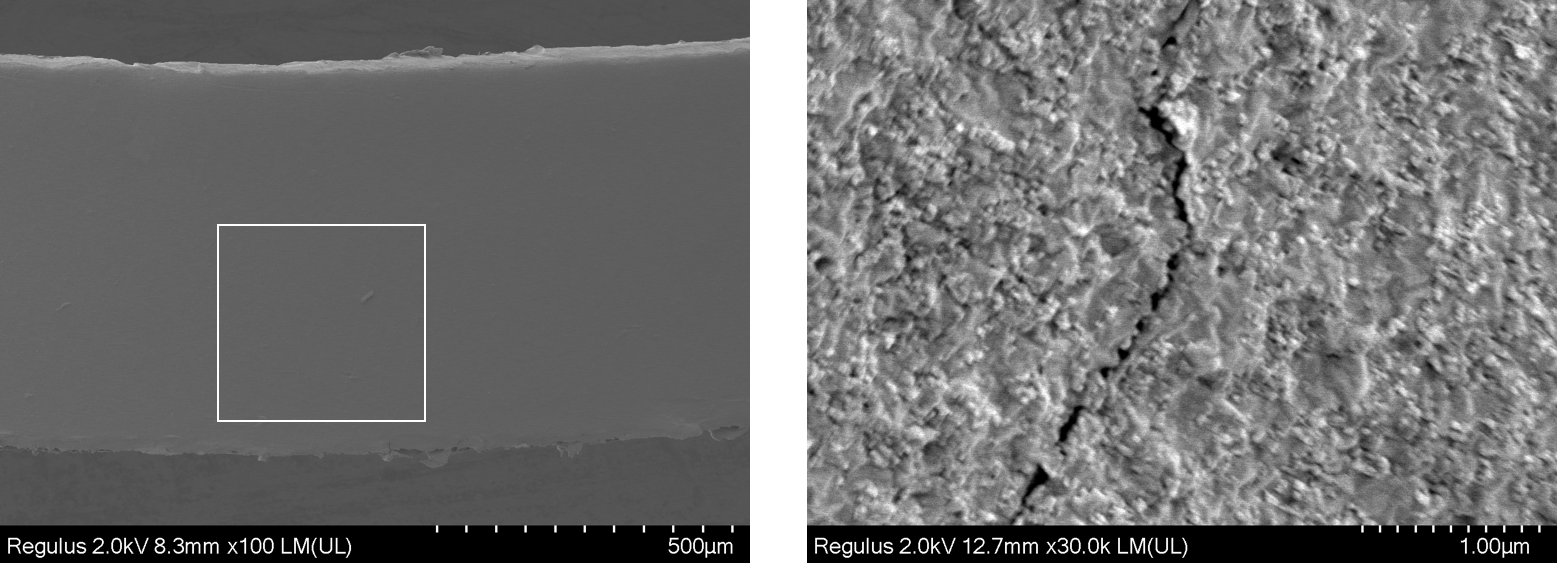


**Figure S16. SEM images.** Electron microscope image of composite material P^2^//**1** during bending.


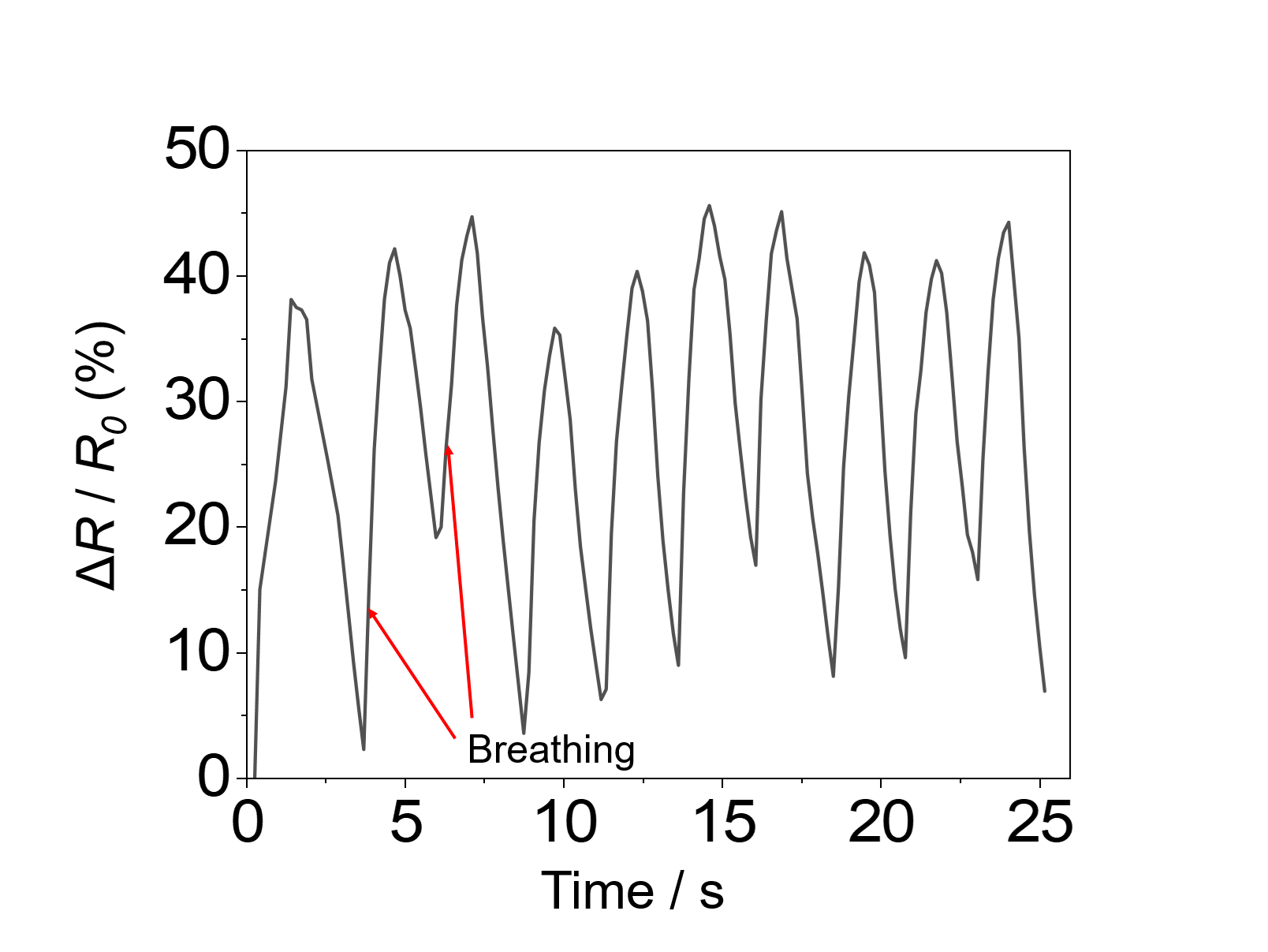


**Figure S17. Resistance changes of the composite material.** The variation in resistance of composite P^2^//**1** over time during the breathing experiment (relative humidity = 30%).


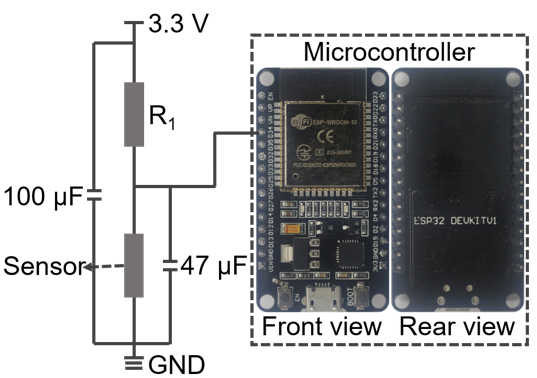


**Figure S18. Cicuit diagram of the sensor and front and rear images.**


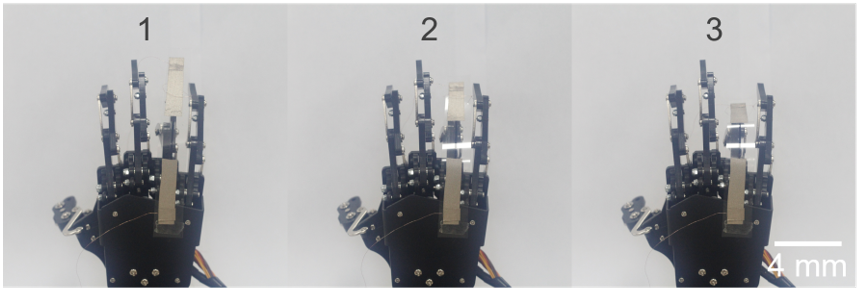


**Figure S19. Sensor bending response.** Photographs of P^2^//**1** crystal sensor bent attached to a bionic finger at different angles.


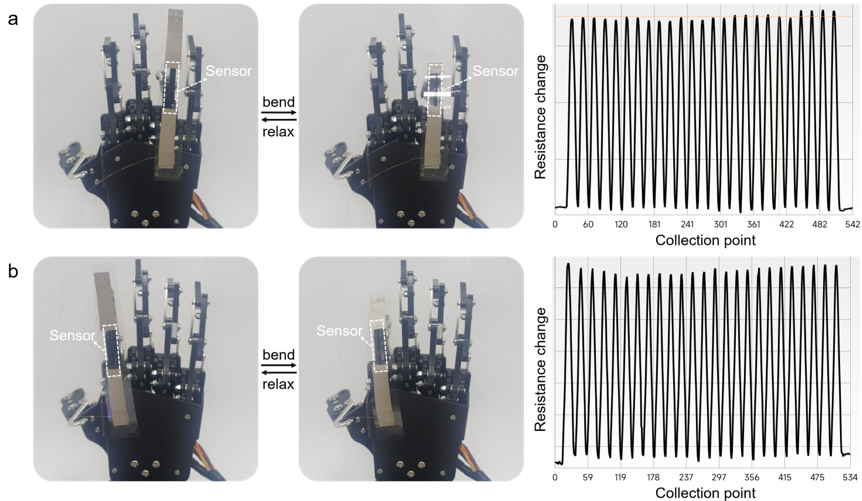


**Figure S20. Motion of a bionic finger monired with a sensor based on the hybrid material.** (a) Photograph of the P^2^//**1** crystal sensor being repeatedly bent on the ring finger and the corresponding change in resistance. (b) Photograph of the P^2^//**1** crystal sensor being repeatedly bent on the index finger and the corresponding change in resistance.


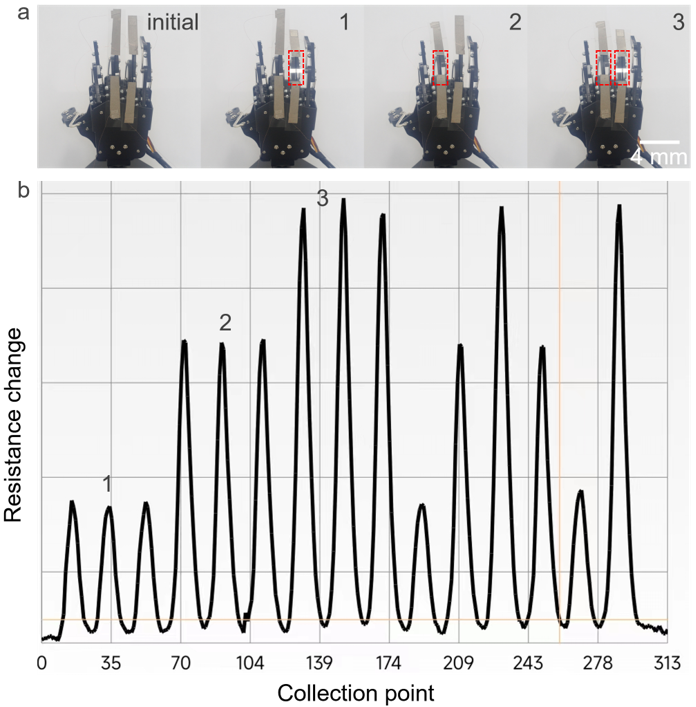


**Figure S21. Finger motion monitoring.** (a) Photographs of different position of bending of a bionic finger. (b) Change in the electrical resistance of P^2^//**1** corresponding to the bending shown in panel a.


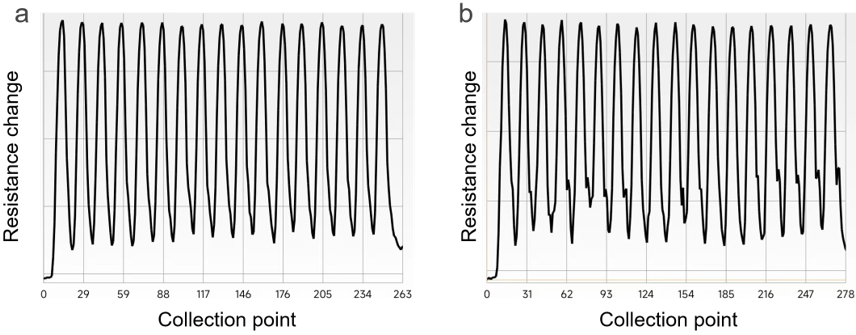


**Figure S22. Durability testing of P^2^//1.** (a) Change in resistance during a bionic finger movement on day 1. (b) Change in resiance on day 7.


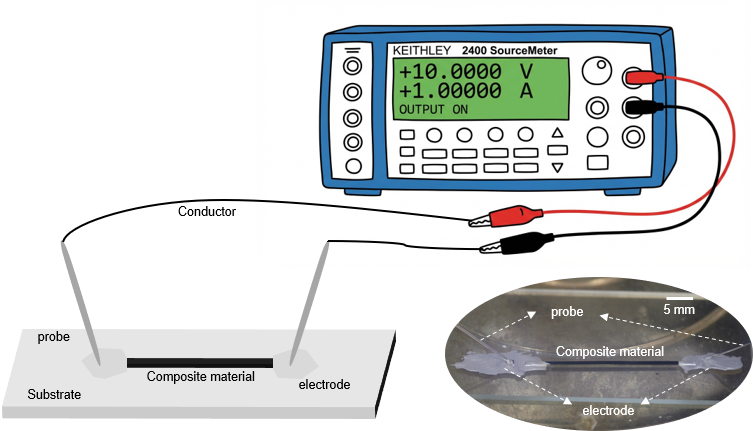


**Figure S23. Electrical testing.** Photographs and schematic diagrams of electrical testing for composite materials.


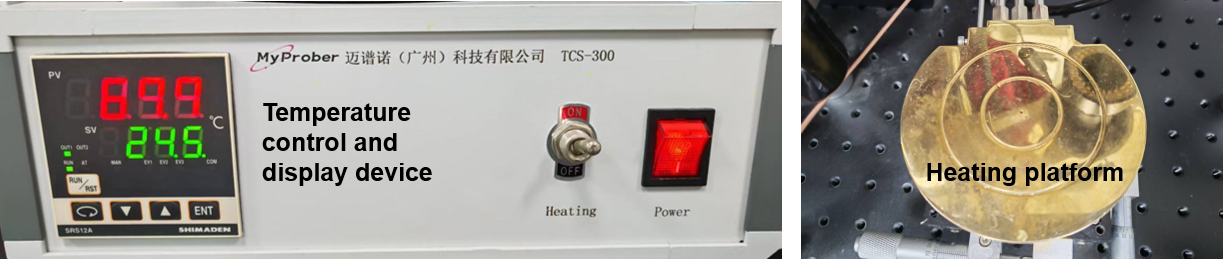


**Figure S24. Experimental setup diagram.** Photograph of the temperature-control setup used for the temperature-dependent measurements, including the temperature control and display device (TCS-300) and the heating platform. The sample was placed directly on the heating platform during the heating test.

**Supporting Tables**

**Table S1.** Length, width, thickness of crystals **1**–**3**

|  | **1** | **2** | **3** |
| --- | --- | --- | --- |
| Length | 1−7 cm | 1−4 cm | 1−3 cm |
| Width | 30−500 μm | 50−300 µm | 50−220 µm |
| Thickness | 20−150 um | 20−130 µm | 10−100 µm |

**Table S2.** Sample labels and their definitions

| **Sample Label** | **Description** |
| --- | --- |
| (PDDA/PSS)_5_//**1**–**3** | A composite sample where the surface of organic crystal **1**–**3** is coated with 5 layers of alternating PDDA and PSS polymers. |
| P^2^//**1**–**3** | A composite sample where the organic crystal **1**–**3** is modified with a PDDA-MXene layer combination on one surface. |

**Table S3.** Fabrication protocol for the preparation of the hybrid crystal-based sensors

| **Step** | **Description** | **Experimental conditions** |
| --- | --- | --- |
| 1 | PDDA adsorption | 1.0 mg mL^−1^, 20 min |
| 2 | Water rinse | 1 min |
| 3 | PSS adsorption | 1.0 mg mL^−1^, 20 min |
| 4 | Water rinse | 1 min |
| 5 | Repetition of PDDA/PSS cycle | Repeated to form 5 bilayers |
| 6 | Final PDDA treatment | 1.0 mg mL^−1^, 20 min |
| 7 | MXene coating | 10 mg mL^−1^ dispersion, syringe-assisted coating |
| 8 | Drying | Ambient conditions |
| 9 | Repetition of PDDA/MXene cycle | Repeated as required for target sensor configuration |

**Table S4.** Summary of sample nomenclature used in this work

| **Sample label** | **Description** |
| --- | --- |
| **1**–**3** | Pristine organic crystal type **1**–**3** |
| (PDDA/PSS)_5_//**1**–**3** | Crystals **1**–**3** coated with five PDDA/PSS bilayers |
| (PDDA/MXene)*_n_*//**1**–**3** | (PDDA/PSS)_5_//**1**–**3** modified with *n* PDDA/MXene deposition cycles |
| P^2^//**1**–**3** | Conductive hybrid crystal derived from crystals **1**–**3** after conductive surface modification |

**Table S5.** Comparison of the operating temperature ranges of the present device with representative MXene-based and crack-based flexible sensors reported in previous studies

| **Reference** | **Material type** | **Operating temperature range** |
| --- | --- | --- |
| This Study | MXene / Organic Crystals | −196 °C to 100 °C |
| Ref 59 | AgNWs / MXene / SEBS | −10 °C to 85 °C |
| Ref 60 | MXene / PU | −10 °C to 80 °C |
| Ref 61 | Crack‑based flexible strain sensor | room temperature |
| Ref 62 | Cracks with network‑crack strain sensor | room temperature |
